# Visible-Light-Active Iodide-Doped BiOBr Coatings for Sustainable Infrastructure

**DOI:** 10.1021/acsami.3c11525

**Published:** 2023-10-12

**Authors:** Mingyue Wang, Raul Quesada-Cabrera, Sanjayan Sathasivam, Matthew O. Blunt, Joanna Borowiec, Claire J. Carmalt

**Affiliations:** † Department of Chemistry, University College London, 20 Gordon Street, London WC1H 0AJ, U.K.; ‡ Department of Chemistry, Institute of Environmental Studies and Natural Resources (i-UNAT, FEAM), Universidad de Las Palmas de Gran Canaria, Campus de Tafira, Las Palmas 35017, Spain; § School of Engineering, London South Bank University, London SE1 0AA, U.K.

**Keywords:** iodide-doped BiOBr thin films, visible-light photocatalysis, resazurin ink, aerosol-assisted chemical vapor deposition (AACVD), self-cleaning windows

## Abstract

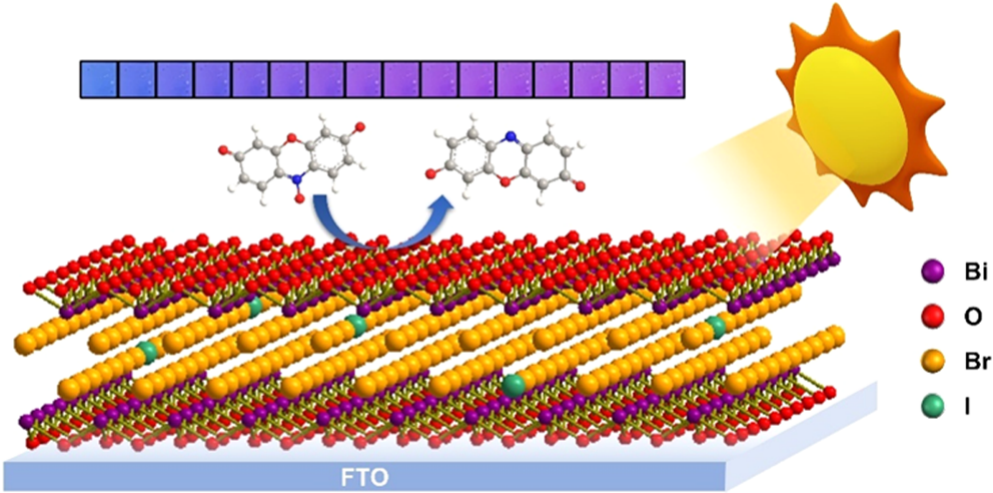

The search for efficient materials for sustainable infrastructure is an urgent challenge toward potential negative emission technologies and the global environmental crisis. Pleasant, efficient sunlight-activated coatings for applications in self-cleaning windows are sought in the glass industry, particularly those produced from scalable technologies. The current work presents visible-light-active iodide-doped BiOBr thin films fabricated using aerosol-assisted chemical vapor deposition. The impact of dopant concentration on the structural, morphological, and optical properties was studied systematically. The photocatalytic properties of the parent materials and as-deposited doped films were evaluated using the smart ink test. An optimized material was identified as containing 2.7 atom % iodide dopant. Insight into the photocatalytic behavior of these coatings was gathered from photoluminescence and photoelectrochemical studies. The optimum photocatalytic performance could be explained from a balance between photon absorption, charge generation, carrier separation, and charge transport properties under 450 nm irradiation. This optimized iodide-doped BiOBr coating is an excellent candidate for the photodegradation of volatile organic pollutants, with potential applications in self-cleaning windows and other surfaces.

## 1 Introduction

Bismuth oxyhalides, with formula BiOX (X = Cl, Br, and I), have received broad interest in areas such as water splitting, pollutant degradation, and photoelectrochemical sensing,^1,2^ with particular focus as emerging materials in photocatalytic applications.^3,4^ The tetragonal crystal structure of BiOX materials consists of [Bi_2_O_2_]^2+^ layers sandwiched between double X^–^ slabs.^5^ Due to the induced polarization, this layered arrangement promotes the formation of an internal static electric field perpendicular to the layers. The existence of this electric field is thought to favor the separation of photogenerated charge carriers, contributing to improved photocatalytic efficiency.^6^ In addition, since BiOX materials are indirect semiconductors, their band structures also facilitate the separation of photogenerated charge carriers, thus further reducing recombination loss.^7^ Among the three types of bismuth oxyhalides, BiOBr has shown more promising photocatalytic behavior compared to BiOCl and BiOI materials for specific applications due to the favorable charge generation and transport properties of the former.^8−10^ The band gap energy of BiOBr (*E*_bg_ = 2.7 eV) allows for the harvesting of photons in the high-energy end of the visible range (up to 460 nm), which is particularly relevant to solar applications.^10^ BiOI has the smallest band gap energy (*E*_bg_ = 1.8 eV) among bismuth oxyhalides, with photon absorption across the entire visible range; however, its narrow band gap accompanies poor redox abilities and also promotes rapid charge recombination.^11,12^ On the other hand, BiOCl (*E*_bg_ > 3.2 eV) can barely absorb photons in the visible range, and thus, it is less suitable for solar applications.

Different material strategies have been recently explored to enhance the photocatalytic properties of BiOBr systems, including controlled growth of specific morphologies and active facets^13−15^ or the engineering of heterojunction systems, together with semiconductors such as AgBr, C_3_N_4_, and CdS.^16−18^ An interesting strategy is the formation of solid solutions, such as BiOCl_*x*_Br_1–*x*_ and BiOBr_*x*_I_1–*x*_, which promotes photon absorption at the low-energy end of the visible range.^19−22^ Conveniently, BiOX materials form similar crystal structures and compositions, with Cl^–^ and Br^–^ or Br^–^ and I^–^ showing similar ionic radii (Cl^–^ = 1.67 Å, Br^–^ = 1.82 Å, and I^–^ = 2.06 Å),^23^ allowing for the continuous adjustment of band structures upon doping with minimum formation of detrimental crystal defects.^24^

Chloride- and iodide-doped BiOBr materials have been reported as promising photocatalysts for applications involving organic pollutant decomposition,^25^ photoelectrochemical biosensors,^26^ or carbon dioxide (CO_2_) reduction.^27^ Most of these materials, however, have been produced in powder form from hydro/solvothermal methods,^20,28,29^ which poses a challenge for scale-up and commercialization. Powder photocatalysts can pose additional challenges to their end use, as they may require embedding within a host material or, when used in solution, may require extraction and recovery steps. Therefore, some work has been reported for the thin-film fabrication of doped BiOX by successive ionic layer adsorption and reaction (SILAR), sol-assisted, and drop-casting methods.^25,30,31^ However, in sol-assisted and drop-casting processes, BiOX powders have to be prepared first, and then extra calcination or coating steps are necessary for film deposition. In addition, the SILAR method can only be utilized to form samples of small sizes on the lab-scale due to its complicated steps and high cost. In this work, iodide-doped BiOBr thin films were deposited on substrates directly using aerosol-assisted chemical vapor deposition (AACVD), which is a feasible, scalable, and cheap method for thin-film deposition. The photocatalytic properties of the iodide-doped BiOBr films were investigated using the standard smart ink method based on the reduction of resazurin (Rz) dye.^32^ The influence of iodide concentration on the crystal structure, compositions, morphologies, and optical properties of the deposited films was fully characterized, as well as the impact of iodide loading on their photocatalytic performance under UV (365 nm) and visible light (450 and 627 nm). A significant improvement in the ink degradation rate was observed after doping iodide in BiOBr under visible-light irradiation, with the best performance seen for 2.7 atom % dopant concentration. Photoluminescence spectroscopy and photoelectrochemical measurements were carried out to gain an insight into the potential underlying mechanism for the observed photocatalytic behavior of these materials.

## 2 Experimental Section

### 2.1 Film Fabrication

Stoichiometric amounts of BiBr_3_ and BiI_3_ (total 1 mmol) were dissolved in 25 mL of anhydrous dimethylformamide (DMF) and ultrasonicated for 10 min to ensure the mixing of precursors. An AACVD setup was used for the film deposition, where a flask containing the precursor solution with a humidifier was attached to the CVD reactor and the generated mist was carried into the reactor under compressed air flow (1.0 l min^–1^). The AACVD process was carried out until the precursor solution was exhausted. Iodide-doped BiOBr_*x*_I_1–*x*_ films were grown on FTO glass (NSG TEC 15) substrates (15 cm × 4.5 cm) at 300 °C for 60 min. Substrates were left to cool to room temperature under air flow.

For comparison, parent materials, BiOBr and BiOI, were deposited under the same conditions from BiBr_3_ (1 mmol) and BiI_3_ (1 mmol), respectively, in anhydrous DMF (25 mL), following a literature procedure.^9^

### 2.2 Physical Characterization

Grazing-incidence X-ray diffraction (GIXRD) patterns were obtained between 5 and 60° (2θ) (0.05° steps, 1.5 s/step) using a Panalytical Empyrean diffractometer, with Cu Kα radiation (λ = 1.5406 Å) at 40 kV and 40 mA emission current. The angle of the incident beam was 1°. Rietveld refinement of XRD data was carried out using MDI Jade 6. The film morphology was studied using a JEOL JSM-7600 field emission scanning electron microscope (SEM) and energy-dispersive spectroscopy (EDS) system. UV–vis–NIR transmission spectra were measured within 300–1100 nm using a Shimadzu UV-3600i Plus spectrometer. X-ray photoelectron spectroscopy (XPS) was carried out using a Thermo Scientific K*-*Alpha spectrometer with monochromated Al Kα_1_ radiation (λ = 8.3418 Å). A dual beam system was employed for charge compensation. Survey scans (0–1200 eV) were obtained at 50 eV pass energy. Depth profiling was performed using an Ar^+^ ion beam for surface etching. All peak positions were calibrated to adventitious carbon (284.8 eV) by using the software CasaXPS. Photoluminescence (PL) spectra were obtained at room temperature using a Renishaw RM1000 spectrometer with an excitation wavelength of 325 nm. Atomic force microscopy (AFM) was performed by using a Keysight 5500 scanning probe microscope. Images over a projected area (5 μm × 5 μm) were recorded in tapping mode using a Si cantilever (NuNano SCOUT-70) with a resonant frequency of ∼70 kHz and ∼2 N m^–1^ spring constant. The roughness factor was obtained by dividing the measured surface area by the projected area.

### 2.3 Functional Test

The photocatalytic properties of the films were assessed using the smart ink test upon evaluation of kinetics in the transformation of resazurin (Rz) to resorufin (Rf) under illumination.^32^ The ink consists of an aqueous hydroxyethyl-cellulose solution containing the redox dye Rz and a sacrificial electron donor (typically glycerol). Positive holes generated upon photoexcitation of the film react with glycerol, while the blue Rz dye is reduced irreversibly to pink Rf by photogenerated electrons. In this work, the Rz ink was prepared following a reported recipe.^33^ 0.006 mmol of hydroxyethyl-cellulose was dissolved in 98.5 mL of distilled water under stirring conditions for 8 h. 0.53 mmol of resazurin sodium salt and 144.78 mmol of glycerol were added to the aqueous polymer and then stirred overnight. The prepared ink was stored in the refrigerator and fully mixed before use. After preirradiation of the films (1.5 cm × 1.5 cm) under UVC light for 10 min, 0.5 mL of the ink was spin-coated on the surface at 6000 rpm for 10 s.

The photocatalytic testing was carried out using a board containing a set of LED lights ranging from 365 to 627 nm (Figure S1).^34^ In this work, top-down irradiation was carried out at 365 nm (2.87 mW cm^–2^), 450 nm (2.01 mW cm^–2^), and 627 nm (1.32 mW cm^–2^). Average RGB values across the film area were recorded as a function of irradiation time from digital images using ImageJ software, where the normalized red component (*R*_t_*=* RGB_R,t_/(RGB_R,t_ + RGB_G,t_ + RGB_B,t_)) was used to monitor the color change in the Rz ink.^35^

### 2.4 Photoelectrochemical Measurements

Transient photocurrent was collected in a three-electrode configuration with an applied voltage of 1.0 *V*_RHE_ at room temperature and under intermittent irradiation. A 220 W Xe lamp with a 420 nm cutoff filter was used as the light source. The electrolyte was an aqueous 0.5 M Na_2_SO_4_ solution at pH 6.6. The deposited iodide-doped BiOBr film was used as the working electrode with Ag/AgCl/3M-KCl and Pt mesh acting as reference and counter electrodes, respectively. An electrochemical workstation (IVIUMSTAT, Netherland) was implemented to apply voltages and measure currents. In the same system, current density–voltage (*J–V*) curves were collected by sweeping the voltage from 0.1 to 1.1 *V*_RHE_ at a rate of 10 mV s^–1^. The applied voltage in our current density–voltage curves was reported against the reversible hydrogen electrode (*V*_RHE_), upon conversion using the Nernst equation *V*_RHE_ = *V*_Ag/AgCl_ + 0.05916 pH + *V*_Ag/AgCl_^⌀^, where *V*_Ag/AgCl_ is the applied potential versus the Ag/AgCl reference electrode and *V*_Ag/AgCl_^⌀^ is the standard reference potential (0.197 *V*_NHE_ at 25 °C).

## 3 Results and Discussion

### 3.1 Synthesis of Iodide-Doped BiOBr Films and Dopant (Iodide) Distribution

Iodide-doped BiOBr films were grown via AACVD from mixtures of BiBr_3_ and BiI_3_ with predetermined molar ratios of 9:1, 8:2, 7:3, 6:4, 5:5, 4:6, and 3:7. Henceforth, the obtained samples will be denoted as **I1-BB**, **I2-BB**, **I3-BB**, **I4-BB**, **I5-BB**, **I6-BB**, and **I7-BB**, respectively, where the number represents the amount of iodine added to the precursor solution per 10 halide anions. During the formation of BiOX solid solutions, iodide was more difficult to be doped into the lattice structure compared with other halides due to its larger radius.^21^ In addition, in the AACVD process, it is relatively hard to form a mist from DMF solutions containing BiI_3_, leading to loss of the iodide source. Therefore, the doping efficiency, as determined from XPS analysis of the halide concentrations in the as-deposited films, was roughly a factor of 10 lower for the BiI_3_ source as compared with the BiBr_3_ source. Interestingly, once the ratio of BiBr_3_ and BiI_3_ in precursor solutions reached above 3:7, such as 2:8 and 1:9, the amount of iodide increased dramatically and finally dominated in the deposited films with poor catalytic performance. Therefore, only pure BiOI and doped films fabricated from precursors with BiI_3_ less than 70% were investigated further.

**Figure 1:**
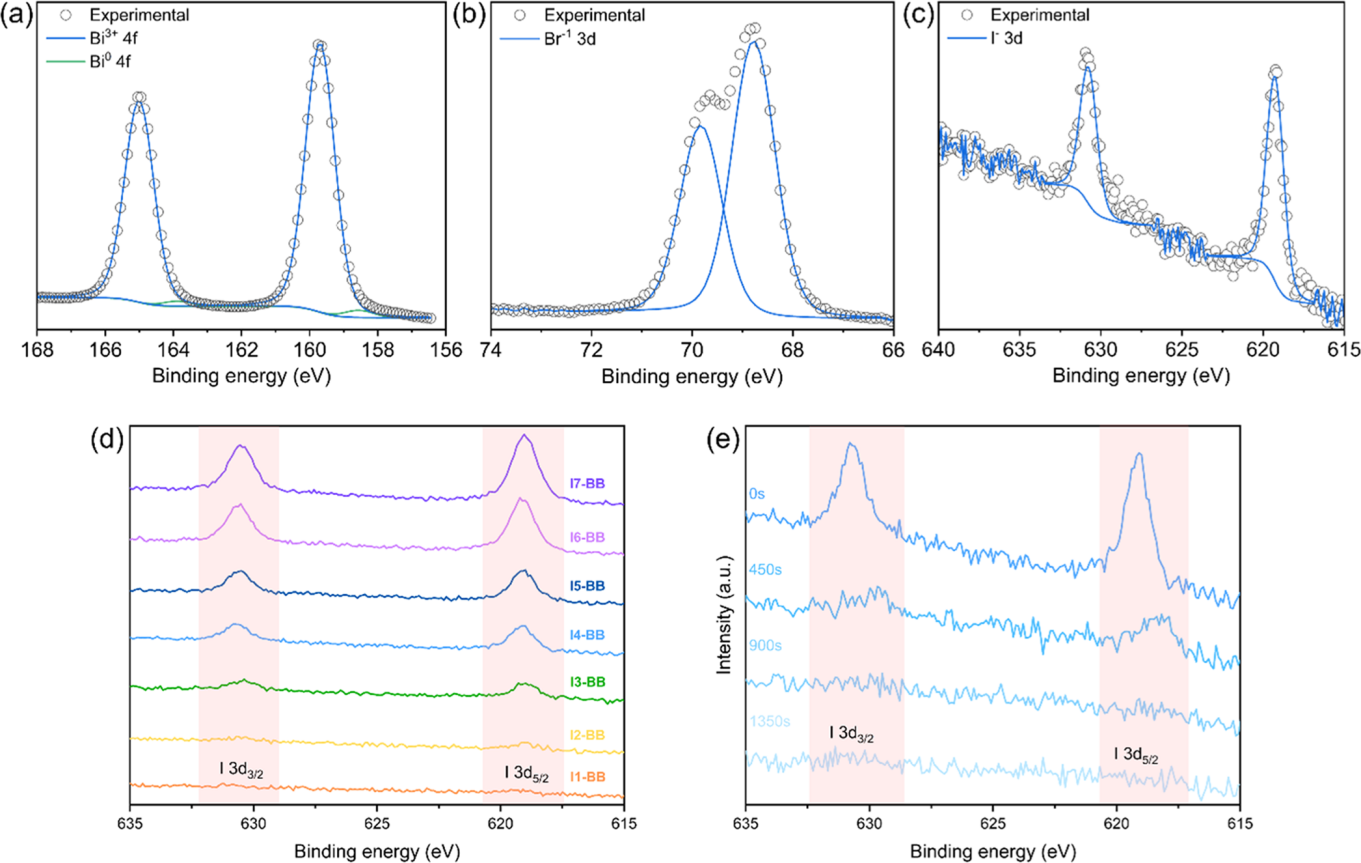
High-resolution XPS spectra of (a) Bi 4f, (b) Br 3d, and (c) I 3d from the **I4-BB** film grown via AACVD. (d) High-resolution XPS spectra of I 3d from all **I-BB** samples. (e) XPS depth profile analysis of I 3d from the **I4-BB** film on FTO.

Due to heavy peak overlap between the Sn Lα from the substrate and the I Lα from the films, EDS was not possible to obtain accurate atomic ratios in films. Therefore, surface composition of the films and elemental valence states were studied using high-resolution XPS. For parent BiOBr and BiOI materials, besides Bi and O elements in both, Br and I elements were observed separately, with peak positions comparable to previous reports observed (Figures S2 and S3).^9,36^ In all doped samples, Bi, Br, O, and I elements coexisted. The Bi 4f spectra were best fitted with a doublet of doublets. The primary Bi 4f_7/2_ was centered at 159.6 (±0.1) eV and assigned to Bi^3+^, while the secondary Bi 4f_7/2_ peak at 158.5 (±0.3) eV closely matched metallic bismuth that often presents itself in XPS due to the photoreduction of BiOX under incident X-rays (Figure 1a).^9,37^ The Br 3d signals were best fitted using a doublet with the 3d_5/2_ centered at 68.8 (±0.1) eV, matching Br^–^ (Figure 1b). As for the high-resolution XPS spectra of I 3d (Figure 1c), the signal produced I 3d_5/2_ peaks centered at 619.2 (±0.3) eV, indicating the successful doping of I^–^ into the BiOBr films.^9,38^

An enhancement in the I 3d peak intensity was clearly observed due to the increasing iodide content in the films (Figure 1d). Atomic ratios of Br and I in **I-BB** films were calculated from the high-resolution XPS Br 3d and I 3d signals, as summarized in Table 1.

**Table 1:** Halide Atomic Concentrations in As-Fabricated **I-BB** Films on FTO

	**I1-BB**	**I2-BB**	**I3-BB**	**I4-BB**	**I5-BB**	**I6-BB**	**I7-BB**
Br	99.3%	99.0%	98.1%	97.3%	96.7%	94.7%	93.7%
I	0.7%	1.0%	1.9%	2.7%	3.3%	5.3%	6.3%

XPS depth profiling studies showed that the peak intensity from I 3d decreased dramatically with increasing etch time, thus demonstrating that iodide was surface segregated (Figure 1e), which is advantageous for heterogeneous photocatalysis as this is a surface-based process.

### 3.2 Structural Characterization of Iodide-Doped BiOBr Films

**Figure 2:**
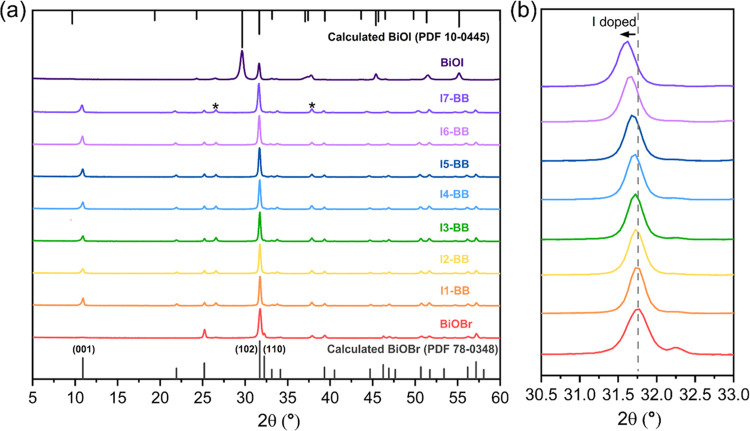
(a) GIXRD analysis of **I-BB** films with increasing concentration of iodide dopant (from bottom to top, molar ratios of 0.7, 1.0, 1.9, 2.7, 3.3, 5.3, and 6.3). The patterns of parent BiOBr and BiOI materials were included for reference. The symbols (*) highlight peaks due to the FTO substrate. (b) Selected region highlighting the shift of the (102) peak upon increasing dopant content.

The structural properties of the iodide-doped BiOBr films were first determined by GIXRD (Figure 2a). The patterns of the parent materials, BiOBr and BiOI, corresponded to pure tetragonal phases (PDF 78-0348 and PDF 10-0445, respectively). The incorporation of iodide in the films resulted in a gradual shift of the (102) peak toward smaller angles (Figure 2b), due to the larger ion radius of I^–^ (2.02 Å) compared to that of Br^–^ (1.82 Å).^23^ This shift was indicative of the formation of a solid solution rather than a simple mixture of the parent phases. Within the dopant concentration range explored in our work, all samples exhibited the tetragonal phase of BiOBr, which further confirmed the doping of iodide in the **I-BB** films. Unit cell parameters and average crystallite sizes of the deposited films were determined from XRD data (Table S1). It can be seen that the introduction of iodide in the structure has significant impact on the *c*-lattice axis, which is consistent with the literature.^39^ The increasing trend in *c* values showed a slight deviation from Vegard’s law (Figure S4), which could be attributed to the intrinsic difference in the halide anionic radii and weak van der Waals forces between adjacent layers. The preferential surface segregation of iodine over bromine, as determined by XPS depth profile analysis, may also be responsible for this deviation.

**Figure 3:**
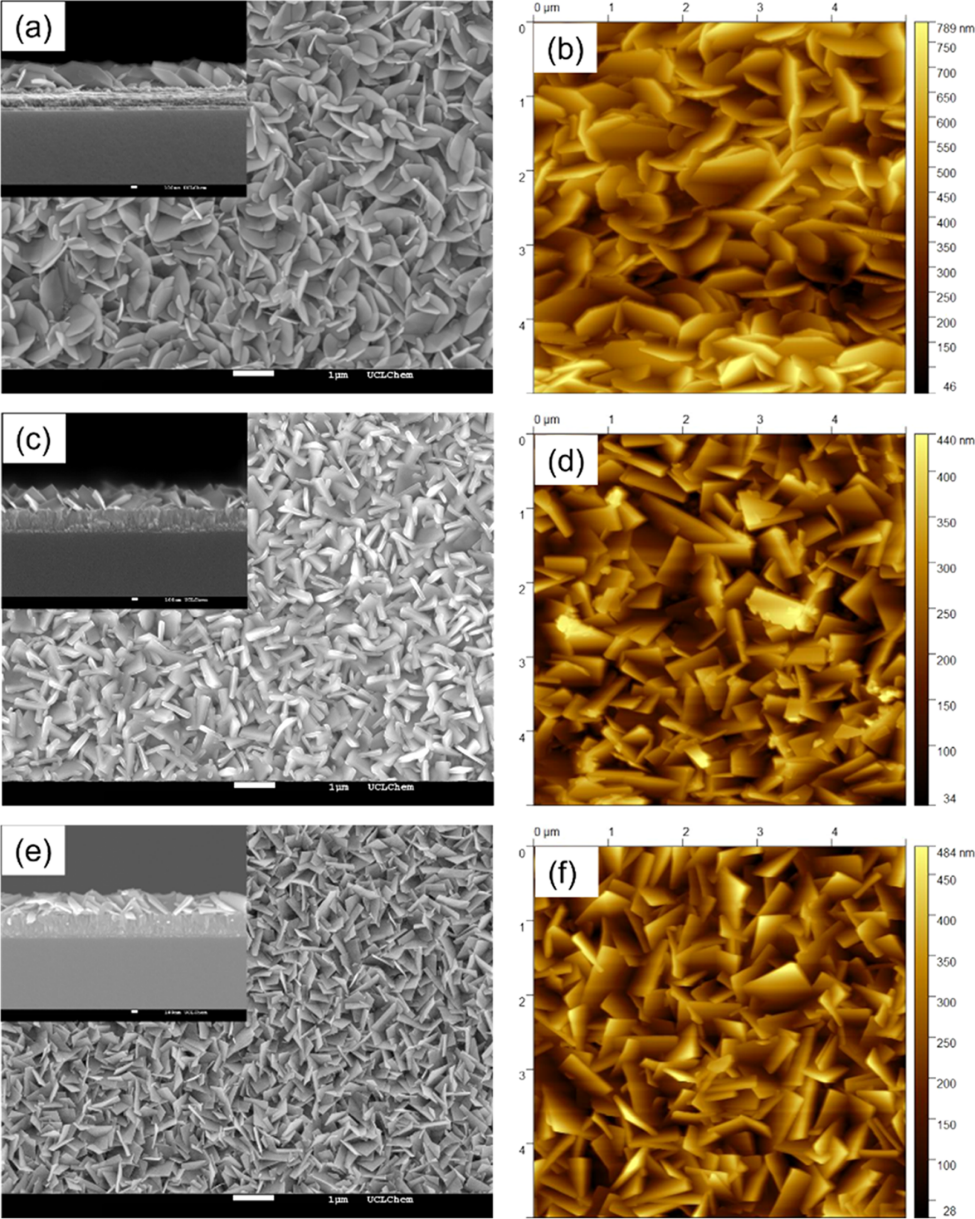
Top-down SEM and inserted cross-sectional SEM images of (a) BiOBr, (c) **I4-BB**, and (e) BiOI. AFM images of (b) BiOBr, (d) **I4-BB**, and (f) BiOI films. All images were of a 5 μm × 5 μm square area.

Top-down SEM was used to study the morphology of the film surface (Figures 3 and S5). All deposited films featured compact and uniform plate-shaped grains. These square nanoplatelets were commonly observed in the investigations of BiOX materials.^9,40,41^ Considering the smaller calculated average crystallite size (Table S1), the particle size observed in top-down SEM images indicated that nanograins in the as-fabricated films were constituted of multiple crystallites. Significant morphological differences were observed among the BiOBr and **I-BB** samples. The grain edge became sharper with more iodide doped. In addition, the grain size of the films decreased from ∼1.3 to ∼0.6 μm. A similar trend was also reported in other iodide-doped BiOX.^21^ All films showed a similar thickness of around 400 nm.

As determined by AFM, the roughness factors of the films were ∼2.03, ∼ 1.67, and ∼1.76, respectively for BiOBr, **I4-BB**, and BiOI films deposited on FTO (Figure 3). The AFM images of other doped samples are also shown in Figure S6. Calculated roughness factors of film surfaces ranged from 2.03 to 1.48, as summarized in Table S2. It was observed that the surface roughness decreased slightly with the increased dopant amount. This likely resulted from the more compact film growth induced by smaller grains, which was shown in the SEM images as well. Greater surface roughness and nanostructuring of films are possible to enhance the photocatalytic activity. However, the roughest BiOBr film did not exhibit the best performance in visible-light photocatalysis, and the change trend of photocatalytic activity, which is discussed later, is not in accordance with that of the roughness factors, indicating that the dramatic difference in visible-light photocatalytic activity could not be attributed to the marginal difference in the samples’ surface areas.

### 3.3 Optical Properties of Iodide-Doped BiOBr Films

**Figure 4:**
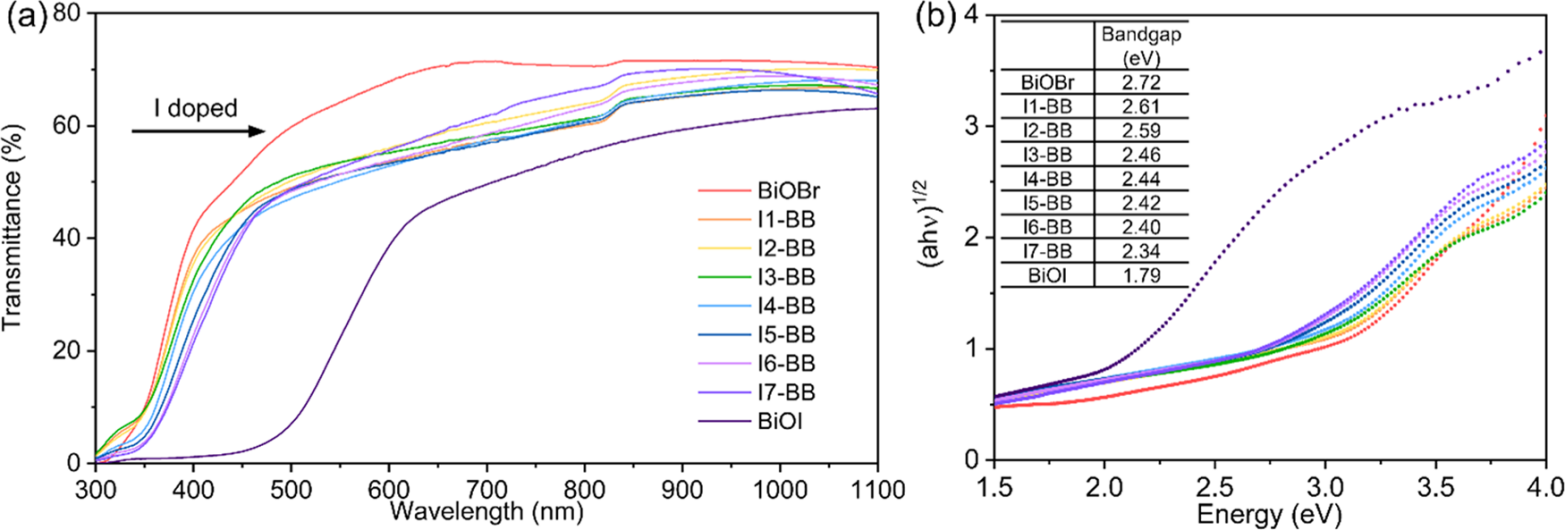
(a) Transmittance spectra of BiOBr, BiOI, and **I-BB** films deposited on FTO. (b) Indirect allowed optical band gap transitions were calculated through the Tauc plot method.

The color of the as-deposited **I-BB** films changed from white to yellow with increasing iodide content, evidenced by a gradual red shift of the absorption edge of the films from 400 to 475 nm (Figure 4a). The sharp edge of pure BiOBr at 400 nm was attributed to an intrinsic transition from the valence band to the conduction band.^42^ Upon doping, however, the absorption curves of the films showed long tails, as it corresponds to the formation of impurity levels in the forbidden band of doped semiconductors.^43,44^ The red shift of the absorption onset involved a significant decrease in band gap energy from 2.72 to 2.34 eV upon incorporation of iodide, as estimated from Tauc plot approximations (Figure 4b).^20^ The regular decreasing trend determined that the band gap could be tailored through adjusting the doping content in the films.

Computational analysis of BiOX materials has shown that the valence band maximum (VBM) is largely composed of X np states, and the conduction band minimum (CBM) is mainly due to the Bi 6p state with minor contributions from X np states.^7,39,45^ Therefore, while uplift of the VBM and downward shift of the CBM would be induced simultaneously during the doping process, the VBM is affected more significantly by the introduction of iodide. As a result, even a trace amount of I^–^ dopant can drastically impact the VBM position leading to a reduced band gap.^46^

### 3.4 Photocatalysis Test

The photocatalytic behavior of the as-prepared **I-BB** films was investigated following the smart Rz ink test under UV (365 nm) and visible light (450 and 627 nm). The Rz-to-Rf transformation was monitored through a digital photographic method from digital images of the Rz ink coated on films and recorded as a function of irradiation time.

**Figure 5:**
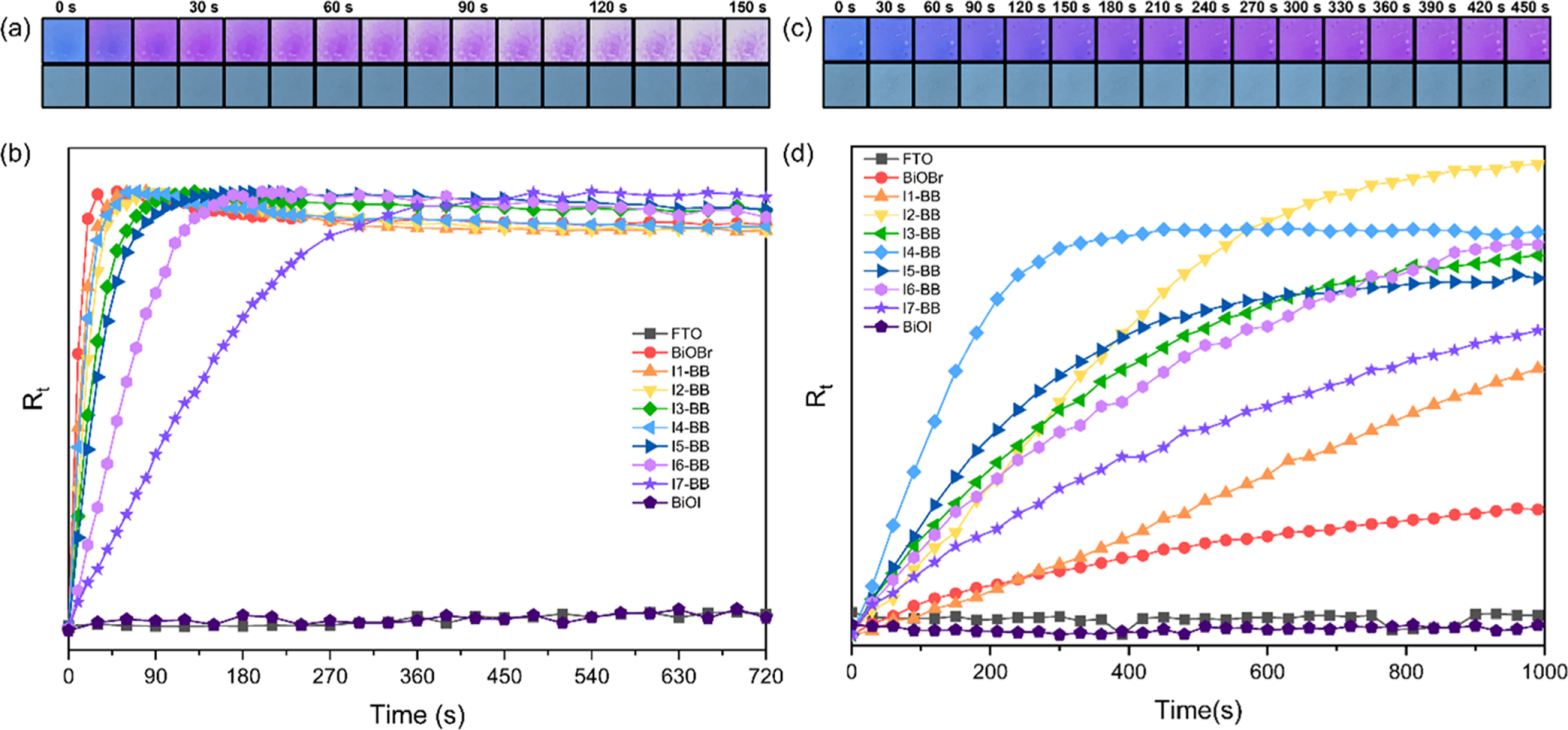
(a) Images of the ink coating on BiOBr (upper) and FTO (lower) under 365 nm irradiation. (b) *R*_t_ vs *t* plots of the smart Rz ink on BiOBr, BiOI, and **I-BB** films deposited on FTO under 365 nm irradiation. (c) Images of the ink coating on **I4-BB** (upper) and FTO (lower) under 450 nm irradiation. (d) *R*_t_ vs *t* plots of the smart Rz ink on BiOBr, BiOI and **I-BB** films deposited on FTO under 450 nm irradiation.

**Figure 6:**
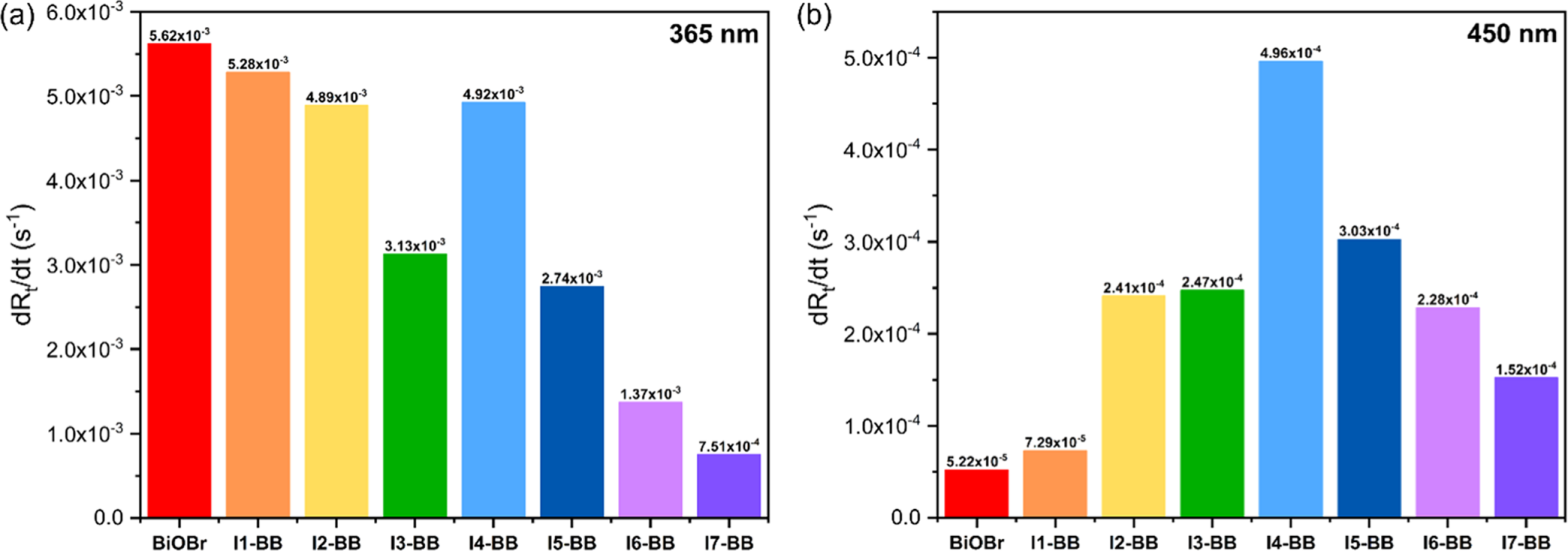
Rates of Rz conversion (d*R*_t_/d*t*) of BiOBr and **I-BB** films deposited on FTO under the irradiation of (a) 365 and (b) 450 nm.

The photocatalytic activity of the samples under UV light was monitored from digital images of the Rz ink up to 720 s at an initial interval of 10 s (up to 240 s), followed by an interval of 30 s. No transformation of the Rz dye was observed in the absence of a photocatalyst (Figure 5a). A rapid color change over the initial irradiation period (150 s) was observed in pure BiOBr, followed by a gradual decrease in kinetics upon I^–^ loading (Figure 5b). From the results of calculated rates of Rz conversion (d*R*_t_/d*t*), it was found that this variation trend was nearly linear with the exception of sample **I4-BB**, which showed an unusual peak in d*R*_t_/d*t* (Figure 6a).

The test under 450 nm irradiation showed a very different scenario. Digital images were obtained for up to 990 s with intervals of 30 s. Figure 5c shows selected images of a representative **I4-BB** sample over an initial irradiation period of 450 s. It was observed that the ink-coated **I4-BB** film followed a smooth color change from blue to pink, while no color change was observed for the ink in the absence of a photocatalyst (Figure 5c). The blank experiment on an FTO substrate showed that the ink was stable, and there was no sign of photolysis under these irradiation conditions. Photosensitization of the film was also ruled out given the peak of the absorption spectrum of the ink at ca. 600 nm (Figure S7). Thus, the color change of the ink was only attributed to the photocatalytic behavior of the films. This assumption was confirmed from photocatalytic tests under 627 nm, where the ink slowly bleached in the absence and presence of bismuth oxyhalides, indicating that the self-degradation of the ink occurred through photolysis under those irradiation conditions (Figure S8).^47^

Derived from recorded images, a series of *R*_t_ values vs irradiation time *t* could be obtained (Figure 5d). From inspection of the figure, sample **I4-BB** (2.7 atom % I^–^ doping) showed the fastest color change, reaching a plateau at around 300 s under 450 nm illumination. The trend of kinetics reaches a maximum with this sample, gradually increasing from pure BiOBr to **I4-BB** and decreasing upon further I^–^ loading. Pure BiOI showed no activity under these conditions, which was attributed to an ultrarapid recombination of charge carriers in this material. The remarkable photocatalytic activity of **I4-BB** exemplified from such a rapid photodegradation of the Rz dye was significantly superior to that of commercial photocatalysts, such as TiO_2_, WO_3_, and C_3_N_4_, and comparable to CdS and BiOCl/Plaster of Paris composites, even under the light source with a lower power density.^47,48^

In order to compare the ink degradation rates of all samples intuitively and quantitatively, d*R*_t_/d*t* was calculated (Figure 6b) and is summarized in Table 2. With the increased I^–^ amount, the values of d*R*_t_/d*t* improved and then declined, where **I4-BB** was able to degrade the Rz dye at a rate of 4.96 × 10^–4^ s^–1^, showing the optimal performance among all measured films. The pure BiOBr was even one order of magnitude lower (5.22 × 10^–5^ s^–1^) in the Rz conversion rate than **I4-BB**.

**Table 2:** d*R*_t_/d*t* of BiOBr and **I-BB** Films Deposited on FTO Under 450 nm Irradiation

	**BiOBr**	**I1-BB**	**I2-BB**	**I3-BB**	**I4-BB**	**I5-BB**	**I6-BB**	**I7-BB**
d*R*_t_/d*t* (×10^–5^ s^–1^)	5.2	7.3	24.1	24.7	49.6	30.3	22.8	15.2

These observations can be explained by consideration of band shifting upon incorporation of the iodide dopant. Under UV irradiation (3.40 eV), electrons can be excited into the conduction bands (CB) of all of the samples, with BiOBr having the most positive valence band (VB) and thus the highest oxidation ability within the family. The sudden relative increase observed for sample **I4-BB** under UV irradiation suggests there may be other factors influencing the photocatalytic activity of these samples, outside the question of VB edge potential. With a relatively stable CBM position, the uplift of VB edge position upon I^–^ loading will narrow the band gap of the materials, favoring the absorption of low-energy photons but also weakening the oxidation ability of holes in the VB.^49,50^ These two competitive factors led to an optimized performance in **I4-BB** (2.7 atom % I^–^) in the case of the tests under 450 nm.

### 3.5 Photoluminescence and Photoelectrochemical Measurements

**Figure 7:**
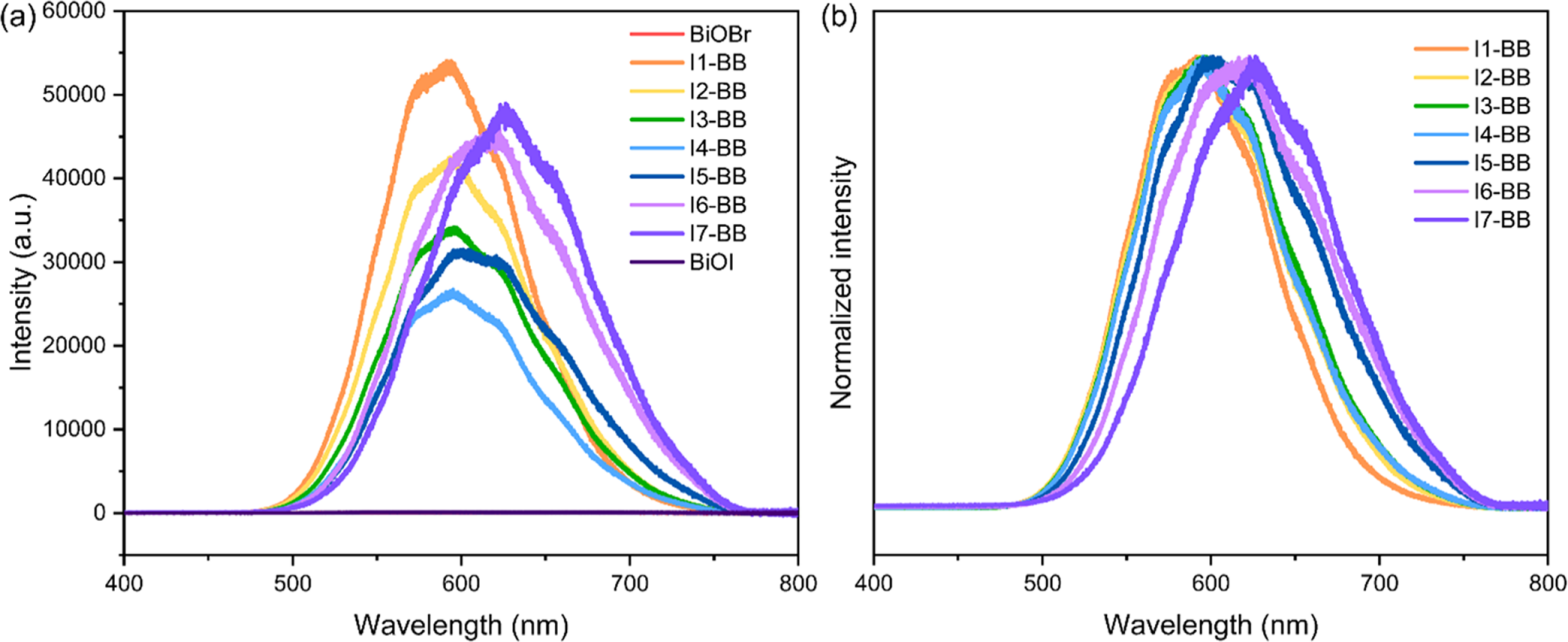
(a) Photoluminescence spectra of BiOBr, BiOI, and **I-BB** films deposited on FTO excited at 325 nm and (b) normalized spectra of **I-BB** films.

Further insight into the photocatalytic properties of the **I-BB** films was gathered from photoluminescence (PL) and photoelectrochemical (PEC) analysis. PL emission results from the recombination of photogenerated electrons and holes after excitation back in the ground state. Thus, the weaker PL emission intensity is widely attributed to less recombination and better separation efficiency of photogenerated electron–hole pairs.^51,52^Figure 7a shows the contrast in PL emission between the **I-BB** samples and the parent materials, BiOBr and BiOI, under UV laser irradiation (λ = 325 nm). As observed, pure BiOBr and BiOI showed no emission peaks, indicating that there was no radiative recombination of photogenerated charge carriers in these materials due to their indirect band features. This observation was in agreement with previous reports in the literature.^53−57^ On the other hand, the impurity states generated by I^–^ doping led to strong PL emission spectra around 600–625 nm in the **I-BB** samples. These spectra were consistent with characteristic emissions from defect states in iodide-doped BiOBr.^15,54^ In addition, the PL band maxima gradually red-shifted upon increasing dopant concentration, as a result of band gap narrowing and increasing photon adsorption (Figure 7b). It is interesting to note that the band intensities decreased upon I^–^ dopant incorporation up to 2.7 atom % (**I4-BB**), where the intensity reached its lowest value (Figure 7a). This was consistent with our previous results during the photocatalytic tests and suggested that **I4-BB** had the lowest carrier recombination rate within the family of **I-BB** materials in this study.

**Figure 8:**
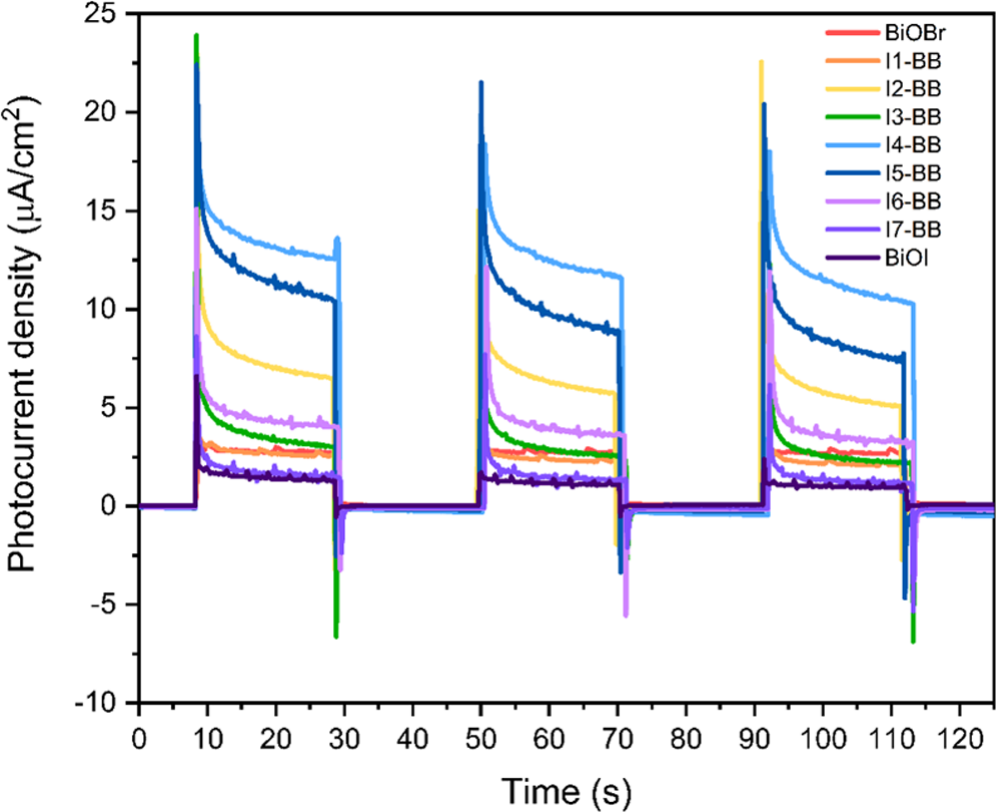
Transient photocurrent response of BiOBr, BiOI, and **I-BB** films deposited on FTO, with the voltage kept at 1.0 V_RHE_. A 220 W Xe lamp with a 420 nm cutoff filter was used as the light source (1 sun illumination). The electrolyte was an aqueous 0.5 M Na_2_SO_4_ solution at pH 6.6.

Furthermore, the separation efficiency of photogenerated charge carriers was investigated by PEC studies. As shown in Figure 8, all of the **I-BB** films and parent materials displayed a stable, reversible and positive transient photocurrent response during three on/off intermittent visible-light irradiation cycles, indicating an effective transfer of charge carriers and successful electron collection for these samples in the PEC cell.^29^ The photocurrent levels remained similar for each sample, demonstrating an excellent photoresponse stability. The figure shows intense photocurrents in the **I-BB** samples compared to the parent materials, following a trend in accordance with that of the photocatalytic behavior of these samples in the smart Rz ink test. The strongest transient photocurrent intensity was recorded for **I4-BB**. This observation confirms an optimum separation efficiency and increased lifetime of photogenerated charge carriers in the **I4-BB** sample.^58−60^ In addition, compared with pure BiOBr, the enhanced photon absorption of the doped samples, shown in the transmittance spectra (Figure 4a), was also one of the reasons for the larger photocurrent response.

Current density–voltage (*J*–*V*) measurements were carried out with voltage swept from 0.13 V_RHE_ to 1.10 V_RHE_ and *J*–*V* curves were obtained under on/off illumination, where the anodic photocurrent increased steadily with the increased voltage (Figure S9). Pure BiOBr and BiOI showed small photoanode currents with onset potentials of ∼0.33 and ∼0.81 V_RHE_, respectively, which could result from the poor absorption of low-energy photons and high charge recombination rates. Nevertheless, the onset potential in **I4-BB** shifted negatively to ∼0.25 V_RHE_ and the current density of this sample (46.5 nA cm^–2^ at 1.0 V_RHE_) increased by almost 10 times compared to pure BiOBr, which was attributed to its fast charge separation and excellent charge transfer ability. It has been reported as well that through the introduction of iodide in BiOBr, the charge transfer resistance of materials could be reduced, leading to higher transfer efficiency of photogenerated charge carriers.^41,61,62^ It is worth noting that the BiOI sample showed photocathodic behavior under cathodic electrode polarization (<0.8 V_RHE_) and photoanodic behavior under anodic electrode polarization (>0.8 V_RHE_) (Figure S9), which is consistent with the observed transient anodic photocurrent response of BiOI with the applied voltage at 1.0 V_RHE_ (Figure 8). This photocurrent switching has been reported for platelet-like BiOI.^63−65^ In bulk semiconductors, photogenerated charge carriers are separated by the electric field of the space charge region, and their transport direction can be determined. The potential drop in the direction perpendicular to the plane of BiOI platelets is considered too small to influence charge transfer due to the small platelet thickness. Therefore, the transfer of photogenerated charge carriers is determined by the electrode–electrolyte interface rather than by the built-in electric field of BiOI. Under cathodic polarization, the conduction band edge of BiOI takes a more negative potential than the redox potential of the electrolyte, which makes the electron transfer from the BiOI electrode to the electrolyte possible. The opposite trend takes place under anodic polarization.^65,66^

Based on the above analysis of PL and PEC measurements, an improved separation and transport behavior of photogenerated charge carriers in the **I-BB** samples, especially **I4-BB**, have been demonstrated compared to those in parent materials. Our results confirmed previous reports in the literature^41,67^ suggesting that hybridization of Br 4p and I 5p orbitals could significantly reduce the mobility of photon-induced holes, whereas the mobility of electrons was rarely influenced, leading to reduced charge recombination. In addition, impurity defects induced by doped iodide may contribute to the trapping of charge carriers in the **I-BB** materials, facilitating charge carrier separation to a certain degree.^68,69^ An excess of dopant sites (beyond 2.7 atom % dopant concentration, **I4-BB** in our case), however, can serve as recombination centers, leading to the detriment of the photocatalytic activity.^43^ The electronic structures of materials are also able to be reconstructed with the increased dopant amount, resulting in the narrowed band gaps while sacrificing the redox ability.^70^ In addition, the potential internal electric field within the intrinsic layered structure of BiOX materials can play an important role in the photocatalytic performance of these materials.^71^ The polarization of the internal electric field is possible to occur due to anionic doping, which is beneficial to the charge transport directed by the electric field force.^22,72^ However, our GIXRD results showed that *c* values increased with I^–^ doping (Table S1), corresponding to an enlarged interlayer space, and thus a longer distance for the charge transport, against the efficient separation of photogenerated charge carriers.^73,74^ As a result, synergistically influenced by the above factors, the charge separation and transport efficiency of iodide-doped BiOBr was improved and maximized when the I^–^ concentration reached 2.7 atom %, making for the optimized visible-light photocatalytic performance.

## 4 Conclusions

Iodide-doped BiOBr (**I-BB**) thin films were successfully deposited in a single step on FTO glass substrates by aerosol-assisted chemical vapor deposition (AACVD). Dopant levels ranged from 0.7 to 6.3 atom %, as confirmed by XPS. Structural analysis was carried out by GIXRD, SEM, and AFM. The photocatalytic properties of the **I-BB** films were explored using the smart ink test, based on the photodegradation of resazurin dye under UV and visible light (LED source). An optimum photocatalytic performance in the visible range (450 nm) was observed for the sample containing 2.7 atom % dopant concentration. This optimized behavior was attributed to a balance between an enhanced photon absorption from band gap narrowing and improved charge carrier separation and transfer ability, based on photoluminescence and photoelectrochemical measurements. The methodology employed in the synthesis of these visible-light-active coatings is a step forward in the engineering of efficient materials for sustainable infrastructure. The pleasant yellow color of these **I-BB** coatings is attractive for their use on self-cleaning windows, for instance, acting on volatile organic pollutants under sunlight.

## Supporting Information Available

Emission spectra of the 365 nm, 450 and 627 nm LED; high-resolution XPS spectra of BiOBr and BiOI films; unit cell parameters, cell volume and average crystallite size of BiOBr, BiOI, and **I-BB** films; comparison of *c* values of **I-BB** samples; top-down and cross-sectional SEM images of BiOBr, **I-BB**, and BiOI films; AFM images of **I-BB** films; roughness factors of BiOBr, **I-BB**, and BiOI films; the absorption spectrum of the Rz smart ink coated on blank FTO; images of the ink coating on **I5-BB** and FTO under 627 nm irradiation; current density–voltage curves of BiOBr, **I4-BB**, and BiOI films on FTO (PDF)

## References

[ref1] Di J.; Xia J.; Li H.; Guo S.; Dai S. Bismuth Oxyhalide Layered Materials for Energy and Environmental Applications. Nano Energy 2017, 41, 172–192. 10.1016/j.nanoen.2017.09.008.

[ref2] Wang H.; Zhang B.; Tang Y.; Wang C.; Zhao F.; Zeng B. Recent Advances in Bismuth Oxyhalide-Based Functional Materials for Photoelectrochemical Sensing. TrAC, Trends Anal. Chem. 2020, 131, 116020 10.1016/j.trac.2020.116020.

[ref3] Cheng H.; Huang B.; Dai Y. Engineering BiOX (X = Cl, Br, I) Nanostructures for Highly Efficient Photocatalytic Applications. Nanoscale 2014, 6 (4), 2009–2026. 10.1039/c3nr05529a. 24430623

[ref4] Hussain A.; Hou J.; Tahir M.; Ali S. S.; Rehman Z. U.; Bilal M.; Zhang T.; Dou Q.; Wang X. Recent Advances in BiOX-Based Photocatalysts to Enhanced Efficiency for Energy and Environment Applications. Catal. Rev. 2022, 1–55. 10.1080/01614940.2022.2041836.

[ref5] Bannister F. A. The Crystal-Structure of the Bismuth Oxyhalides. Mineral. Mag. J. Mineral. Soc. 1935, 24 (149), 49–58. 10.1180/minmag.1935.024.149.01.

[ref6] Mi Y.; Zhou M.; Wen L.; Zhao H.; Lei Y. A Highly Efficient Visible-Light Driven Photocatalyst: Two Dimensional Square-like Bismuth Oxyiodine Nanosheets. Dalton Trans. 2014, 43 (25), 9549–9556. 10.1039/C4DT00798K. 24829111

[ref7] Zhang H.; Liu L.; Zhou Z. Towards Better Photocatalysts: First-Principles Studies of the Alloying Effects on the Photocatalytic Activities of Bismuth Oxyhalides under Visible Light. Phys. Chem. Chem. Phys. 2012, 14 (3), 1286–1292. 10.1039/C1CP23516H. 22146949

[ref8] Chen L.; Huang R.; Xiong M.; Yuan Q.; He J.; Jia J.; Yao M.; Luo S.; Au C.; Yin S. Room-Temperature Synthesis of Flower-Like BiOX (X=Cl, Br, I) Hierarchical Structures and Their Visible-Light Photocatalytic Activity. Inorg. Chem. 2013, 52 (19), 11118–11125. 10.1021/ic401349j. 24050663

[ref9] Bhachu D. S.; Moniz S. J. A.; Sathasivam S.; Scanlon D. O.; Walsh A.; Bawaked S. M.; Mokhtar M.; Obaid A. Y.; Parkin I. P.; Tang J.; Carmalt C. J. Bismuth Oxyhalides: Synthesis, Structure and Photoelectrochemical Activity. Chem. Sci. 2016, 7 (8), 4832–4841. 10.1039/C6SC00389C. 30155131 PMC6016733

[ref10] An H.; Du Y.; Wang T.; Wang C.; Hao W.; Zhang J. Photocatalytic Properties of BiOX (X = Cl, Br, and I). Rare Met. 2008, 27 (3), 243–250. 10.1016/S1001-0521(08)60123-0.

[ref11] Ganose A. M.; Cuff M.; Butler K. T.; Walsh A.; Scanlon D. O. Interplay of Orbital and Relativistic Effects in Bismuth Oxyhalides: BiOF, BiOCl, BiOBr, and BiOI. Chem. Mater. 2016, 28 (7), 1980–1984. 10.1021/acs.chemmater.6b00349. 27274616 PMC4887134

[ref12] Gómez-Velázquez L. S.; Hernández-GordilloCatedrático CONACYT A.; Robinson M. J.; Leppert V. J.; Rodil S. E.; Bizarro M. The Bismuth Oxyhalide Family: Thin Film Synthesis and Periodic Properties. Dalton Trans. 2018, 47 (35), 12459–12467. 10.1039/c8dt02642d. 30140815

[ref13] Zhang J.; Shi F.; Lin J.; Chen D.; Gao J.; Huang Z.; Ding X.; Tang C. Self-Assembled 3-D Architectures of BiOBr as a Visible Light-Driven Photocatalyst. Chem. Mater. 2008, 20 (9), 2937–2941. 10.1021/cm7031898.

[ref14] Zhang H.; Yang Y.; Zhou Z.; Zhao Y.; Liu L. Enhanced Photocatalytic Properties in BiOBr Nanosheets with Dominantly Exposed (102) Facets. J. Phys. Chem. C 2014, 118 (26), 14662–14669. 10.1021/jp5035079.

[ref15] Li H.; Shang J.; Ai Z.; Zhang L. Efficient Visible Light Nitrogen Fixation with BiOBr Nanosheets of Oxygen Vacancies on the Exposed {001} Facets. J. Am. Chem. Soc. 2015, 137 (19), 6393–6399. 10.1021/jacs.5b03105. 25874655

[ref16] Kong L.; Jiang Z.; Lai H. H.; Nicholls R. J.; Xiao T.; Jones M. O.; Edwards P. P. Unusual Reactivity of Visible-Light-Responsive AgBr–BiOBr Heterojunction Photocatalysts. J. Catal. 2012, 293, 116–125. 10.1016/j.jcat.2012.06.011.

[ref17] Fu J.; Tian Y.; Chang B.; Xi F.; Dong X. BiOBr–Carbon Nitride Heterojunctions: Synthesis, Enhanced Activity and Photocatalytic Mechanism. J. Mater. Chem. 2012, 22 (39), 21159–21166. 10.1039/c2jm34778d.

[ref18] Cui W.; An W.; Liu L.; Hu J.; Liang Y. Synthesis of CdS/BiOBr Composite and Its Enhanced Photocatalytic Degradation for Rhodamine B. Appl. Surf. Sci. 2014, 319 (1), 298–305. 10.1016/j.apsusc.2014.05.179.

[ref19] Liu Y.; Son W.-J.; Lu J.; Huang B.; Dai Y.; Whangbo M.-H. Composition Dependence of the Photocatalytic Activities of BiOCl_1-x_Br_x_ Solid Solutions under Visible Light. Chem. – Eur. J. 2011, 17 (34), 9342–9349. 10.1002/chem.201100952. 21732448

[ref20] Jia Z.; Wang F.; Xin F.; Zhang B. Simple Solvothermal Routes to Synthesize 3D BiOBr x I 1- x Microspheres and Their Visible-Light-Induced Photocatalytic Properties. Ind. Eng. Chem. Res. 2011, 50 (11), 6688–6694. 10.1021/ie102310a.

[ref21] Zhang B.; Ji G.; Liu Y.; Gondal M. A.; Chang X. Efficient Adsorption and Photocatalytic Pceerformance of Flower-like Three-Dimensional (3D) I-Doped BiOClBr Photocatalyst. Catal. Commun. 2013, 36, 25–30. 10.1016/j.catcom.2013.02.021.

[ref22] Wang Y.; Zhang S.; Yan Y.; Ren H.; Chen J.; Liu L.; Wu X. Multi-Anions-Coupled Electronic States in Cl–-Doped BiOBr Induce Highly Efficient Decomposition of Tetracycline Hydrochloride. Mater. Res. Bull. 2023, 158, 112045 10.1016/j.materresbull.2022.112045.

[ref23] Shannon R. D. Revised Effective Ionic Radii and Systematic Studies of Interatomic Distances in Halides and Chalcogenides. Acta Crystallogr., Sect. A: Cryst. Phys., Diffr., Theor. Gen. Crystallogr. 1976, 32 (5), 751–767. 10.1107/S0567739476001551.

[ref24] Qi L.; Yang Y.; Zhang P.; Le Y.; Wang C.; Wu T. Hierarchical Flower-like BiOI_x_Br_(1-x)_ Solid Solution Spheres with Enhanced Visible-Light Photocatalytic Activity. Appl. Surf. Sci. 2019, 467–468, 792–801. 10.1016/j.apsusc.2018.10.184.

[ref25] Dandapat A.; Horovitz I.; Gnayem H.; Sasson Y.; Avisar D.; Luxbacher T.; Mamane H. Solar Photocatalytic Degradation of Trace Organic Pollutants in Water by Bi(0)-Doped Bismuth Oxyhalide Thin Films. ACS Omega 2018, 3 (9), 10858–10865. 10.1021/acsomega.8b00759. 31459198 PMC6645048

[ref26] Feng J.; Li N.; Du Y.; Ren X.; Wang X.; Liu X.; Ma H.; Wei Q. Ultrasensitive Double-Channel Microfluidic Biosensor-Based Cathodic Photo-Electrochemical Analysis via Signal Amplification of SOD-Au@PANI for Cardiac Troponin i Detection. Anal. Chem. 2021, 93 (42), 14196–14203. 10.1021/acs.analchem.1c02922. 34636556

[ref27] Yin H. Y.; Zheng Y. F.; Song X. C. Synthesis and Enhanced Visible Light Photocatalytic CO 2 Reduction of BiPO 4 – BiOBr x I 1–x p–n Heterojunctions with Adjustable Energy Band. RSC Adv. 2019, 9 (20), 11005–11012. 10.1039/C9RA01416K. 35520253 PMC9063035

[ref28] Deng F.; Luo Y.; Li H.; Xia B.; Luo X.; Luo S.; Dionysiou D. D. Efficient Toxicity Elimination of Aqueous Cr(VI) by Positively-Charged BiOClxI1-x, BiOBrxI1-x and BiOClxBr1-x Solid Solution with Internal Hole-Scavenging Capacity via the Synergy of Adsorption and Photocatalytic Reduction. J. Hazard. Mater. 2020, 383, 121127 10.1016/j.jhazmat.2019.121127. 31518810

[ref29] Qin Q.; Guo Y.; Zhou D.; Yang Y.; Guo Y. Facile Growth and Composition-Dependent Photocatalytic Activity of Flowerlike BiOCl1–xBrx Hierarchical Microspheres. Appl. Surf. Sci. 2016, 390, 765–777. 10.1016/j.apsusc.2016.08.134.

[ref30] Jia H.; Li Y.; Mao Y.; Yu D.; He W.; Zheng Z. Room Temperature Synthesis of BiOBr 1–x I x Thin Films with Tunable Structure and Conductivity Type for Enhanced Photoelectric Performance. RSC Adv. 2020, 10 (68), 41755–41763. 10.1039/D0RA08211B. 35516544 PMC9057841

[ref31] Alansi A. M.; Qahtan T. F.; Saleh T. A. Solar-Driven Fixation of Bismuth Oxyhalides on Reduced Graphene Oxide for Efficient Sunlight-Responsive Immobilized Photocatalytic Systems. Adv. Mater. Interfaces 2021, 8 (3), 2001463 10.1002/admi.202001463.

[ref32] Mills A.; Wang J.; Lee S. K.; Simonsen M. An Intelligence Ink for Photocatalytic Films. Chem. Commun. 2005, (21), 2721–2723. 10.1039/b501131k. 15917932

[ref33] Mills A.; Wells N.; O’Rourke C. Correlation between ΔAbs, ΔRGB (Red) and Stearic Acid Destruction Rates Using Commercial Self-Cleaning Glass as the Photocatalyst. Catal. Today 2014, 230, 245–249. 10.1016/j.cattod.2013.11.023.

[ref34] Casado C.; Timmers R.; Sergejevs A.; Clarke C. T.; Allsopp D. W. E.; Bowen C. R.; van Grieken R.; Marugán J. Design and Validation of a LED-Based High Intensity Photocatalytic Reactor for Quantifying Activity Measurements. Chem. Eng. J. 2017, 327, 1043–1055. 10.1016/j.cej.2017.06.167.

[ref35] Mills A.; O’Rourke C.; Wells N. A Smart Ink for the Assessment of Low Activity Photocatalytic Surfaces. Analyst 2014, 139 (21), 5409–5414. 10.1039/C4AN01375A. 25219346

[ref36] Wang M.; Kafizas A.; Sathasivam S.; Blunt M. O.; Moss B.; Gonzalez-carrero S.; Carmalt C. J. ZnO/BiOI Heterojunction Photoanodes with Enhanced Photoelectrochemical Water Oxidation Activity. Appl. Catal., B 2023, 331, 122657 10.1016/j.apcatb.2023.122657.

[ref37] Jia X.; Cao J.; Lin H.; Zhang M.; Guo X.; Chen S. Transforming Type-I to Type-II Heterostructure Photocatalyst via Energy Band Engineering: A Case Study of I-BiOCl/I-BiOBr. Appl. Catal., B 2017, 204, 505–514. 10.1016/j.apcatb.2016.11.061.

[ref38] Jia X.; Cao J.; Lin H.; Zhang M.; Guo X.; Chen S. Novel I-BiOBr/BiPO 4 Heterostructure: Synergetic Effects of I – Ion Doping and the Electron Trapping Role of Wide-Band-Gap BiPO 4 Nanorods. RSC Adv. 2016, 6 (61), 55755–55763. 10.1039/C6RA06330F.

[ref39] Liu G.; Wang T.; Ouyang S.; Liu L.; Jiang H.; Yu Q.; Kako T.; Ye J. Band-Structure-Controlled BiO(ClBr) (1–x)/2 I x Solid Solutions for Visible-Light Photocatalysis. J. Mater. Chem. A 2015, 3 (15), 8123–8132. 10.1039/C4TA07128J.

[ref40] Gnayem H.; Sasson Y. Hierarchical Nanostructured 3D Flowerlike BiOCl x Br 1– x Semiconductors with Exceptional Visible Light Photocatalytic Activity. ACS Catal. 2013, 3 (2), 186–191. 10.1021/cs3005133.

[ref41] Wang Q.; Liu Z.; Liu D.; Liu G.; Yang M.; Cui F.; Wang W. Ultrathin Two-Dimensional BiOBr_x_I_1-x_ Solid Solution with Rich Oxygen Vacancies for Enhanced Visible-Light-Driven Photoactivity in Environmental Remediation. Appl. Catal., B 2018, 236, 222–232. 10.1016/j.apcatb.2018.05.029.

[ref42] Wang W.; Huang F.; Lin X.; Yang J. Visible-Light-Responsive Photocatalysts XBiOBr–(1–x)BiOI. Catal. Commun. 2008, 9 (1), 8–12. 10.1016/j.catcom.2007.05.014.

[ref43] Lin H.; Li X.; Cao J.; Chen S.; Chen Y. Novel I^–^-Doped BiOBr Composites: Modulated Valence Bands and Largely Enhanced Visible Light Phtotocatalytic Activities. Catal. Commun. 2014, 49, 87–91. 10.1016/j.catcom.2014.02.010.

[ref44] Shen S.; Zhao L.; Zhou Z.; Guo L. Enhanced Photocatalytic Hydrogen Evolution over Cu-Doped ZnIn_2_S_4_ under Visible Light Irradiation. J. Phys. Chem. C 2008, 112 (41), 16148–16155. 10.1021/jp804525q.

[ref45] Huo X.; Huang L.-F. Physical Spread and Technical Upshift in the Band Gaps of Visible-Light Photocatalytic Bismuth Oxyhalide Solid Solutions. Comput. Mater. Sci. 2020, 184, 109870 10.1016/j.commatsci.2020.109870.

[ref46] Kong L.; Guo J.; Makepeace J. W.; Xiao T.; Greer H. F.; Zhou W.; Jiang Z.; Edwards P. P. Rapid Synthesis of BiOBr_x_I_1-x_ Photocatalysts: Insights to the Visible-Light Photocatalytic Activity and Strong Deviation from Vegard’s Law. Catal. Today 2019, 335, 477–484. 10.1016/j.cattod.2019.02.013.

[ref47] Mills A.; Wells N.; O’Rourke C. Probing the Activities of UV and Visible-Light Absorbing Photocatalyst Powders Using a Resazurin-Based Photocatalyst Activity Indicator Ink (Rz Paii). J. Photochem. Photobiol., A 2017, 338, 123–133. 10.1016/j.jphotochem.2017.01.030.

[ref48] Singh V. P.; Mishra D.; Kabachkov E. N.; Shul’ga Y. M.; Vaish R. The Characteristics of BiOCl/Plaster of Paris Composites and Their Photocatalytic Performance under Visible Light Illumination for Self-Cleaning. Mater. Sci. Energy Technol. 2020, 3, 299–307. 10.1016/J.MSET.2019.12.001.

[ref49] Zhang J.; Han Q.; Zhu J.; Wang X. A Facile and Rapid Room-Temperature Route to Hierarchical Bismuth Oxyhalide Solid Solutions with Composition-Dependent Photocatalytic Activity. J. Colloid Interface Sci. 2016, 477, 25–33. 10.1016/j.jcis.2016.05.027. 27236841

[ref50] Zhao R.; Jia Z.; Li T.; Liu J.; Li R.; Wang Y.; Wang Y.; Zhang X.; Fan C. Concise Fabrication of 3D Rose-like BiOBrxI1–x with Exceptional Wide Spectrum Visible-Light Photocatalytic Activity. Inorg. Chem. Commun. 2019, 101, 150–159. 10.1016/j.inoche.2019.01.021.

[ref51] Tebyetekerwa M.; Zhang J.; Xu Z.; Truong T. N.; Yin Z.; Lu Y.; Ramakrishna S.; Macdonald D.; Nguyen H. T. Mechanisms and Applications of Steady-State Photoluminescence Spectroscopy in Two-Dimensional Transition-Metal Dichalcogenides. ACS Nano 2020, 14 (11), 14579–14604. 10.1021/acsnano.0c08668. 33155803

[ref52] Wang X.; Ren Y.; Li Y.; Zhang G. Fabrication of 1D/2D BiPO4/g-C3N4 Heterostructured Photocatalyst with Enhanced Photocatalytic Efficiency for NO Removal. Chemosphere 2022, 287, 132098 10.1016/j.chemosphere.2021.132098. 34509004

[ref53] Wu J.; Xie Y.; Ling Y.; Dong Y.; Li J.; Li S.; Zhao J. Synthesis of Flower-Like g-C_3_N_4_/BiOBr and Enhancement of the Activity for the Degradation of Bisphenol A Under Visible Light Irradiation. Front. Chem. 2019, 7, 649 10.3389/fchem.2019.00649. 31632947 PMC6779780

[ref54] Wang H.; Yong D.; Chen S.; Jiang S.; Zhang X.; Shao W.; Zhang Q.; Yan W.; Pan B.; Xie Y. Oxygen-Vacancy-Mediated Exciton Dissociation in BiOBr for Boosting Charge-Carrier-Involved Molecular Oxygen Activation. J. Am. Chem. Soc. 2018, 140 (5), 1760–1766. 10.1021/jacs.7b10997. 29319310

[ref55] Li J.; Zhou Q.; Yang F.; Wu L.; Li W.; Ren R.; Lv Y. Uniform Flower-like BiOBr/BiOI Prepared by a New Method: Visible-Light Photocatalytic Degradation, Influencing Factors and Degradation Mechanism. New J. Chem. 2019, 43 (37), 14829–14840. 10.1039/C9NJ03038G.

[ref56] Li Y.; Li Z.; Gao L. Construction of Z-Scheme BiOI/g-C_3_N_4_ Heterojunction with Enhanced Photocatalytic Activity and Stability under Visible Light. J. Mater. Sci.: Mater. Electron. 2019, 30 (13), 12769–12782. 10.1007/s10854-019-01642-0.

[ref57] Sun Y.; Zhang W.; Xiong T.; Zhao Z.; Dong F.; Wang R.; Ho W.-K. Growth of BiOBr Nanosheets on C3N4 Nanosheets to Construct Two-Dimensional Nanojunctions with Enhanced Photoreactivity for NO Removal. J. Colloid Interface Sci. 2014, 418, 317–323. 10.1016/j.jcis.2013.12.037. 24461851

[ref58] Bi C.; Cao J.; Lin H.; Wang Y.; Chen S. Tunable Photocatalytic and Photoelectric Properties of I – -Doped BiOBr Photocatalyst: Dramatic PH Effect. RSC Adv. 2016, 6 (19), 15525–15534. 10.1039/C5RA22943J.

[ref59] Fung C. S. L.; Khan M.; Kumar A.; Lo I. M. C. Visible-Light-Driven Photocatalytic Removal of PPCPs Using Magnetically Separable Bismuth Oxybromo-Iodide Solid Solutions: Mechanisms, Pathways, and Reusability in Real Sewage. Sep. Purif. Technol. 2019, 216, 102–114. 10.1016/j.seppur.2019.01.077.

[ref60] Jia X.; Cao J.; Lin H.; Chen Y.; Fu W.; Chen S. One-Pot Synthesis of Novel Flower-like BiOBr_0.9_I_0.1_/BiOI Heterojunction with Largely Enhanced Electron-Hole Separation Efficiency and Photocatalytic Performances. J. Mol. Catal. A: Chem. 2015, 409, 94–101. 10.1016/j.molcata.2015.08.008.

[ref61] Zhang G.; Zhang L.; Liu Y.; Liu L.; Huang C.-P.; Liu H.; Li J. Substitution Boosts Charge Separation for High Solar-Driven Photocatalytic Performance. ACS Appl. Mater. Interfaces 2016, 8 (40), 26783–26793. 10.1021/acsami.6b08676. 27662229

[ref62] Lu R.; Zahid A. H.; Han Q. Insight into the Photocatalytic Mechanism of the Optimal x Value in the BiOBr x I 1– x, BiOCl x I 1– x and BiOCl x Br 1– x Series Varying with Pollutant Type. Nanoscale 2022, 14 (37), 13711–13721. 10.1039/D2NR03726B. 36093962

[ref63] Poznyak S. K.; Kulak A. I. Photoelectrochemical Properties of Bismuth Oxyhalide Films. Electrochim. Acta 1990, 35 (11–12), 1941–1947. 10.1016/0013-4686(90)87103-9.

[ref64] Kwolek P.; Szaciłowski K. Photoelectrochemistry of N-Type Bismuth Oxyiodide. Electrochim. Acta 2013, 104, 448–453. 10.1016/j.electacta.2012.10.001.

[ref65] Kazyrevich M. E.; Malashchonak M. V.; Mazanik A. V.; Streltsov E. A.; Kulak A. I.; Bhattacharya C. Photocurrent Switching Effect on Platelet-like BiOI Electrodes: Influence of Redox System, Light Wavelength and Thermal Treatment. Electrochim. Acta 2016, 190, 612–619. 10.1016/j.electacta.2015.12.229.

[ref66] Gawęda S.; Kowalik R.; Kwolek P.; MacYk W.; Mech J.; Oszajca M.; Podborska A.; Szaciłowski K. Nanoscale Digital Devices Based on the Photoelectrochemical Photocurrent Switching Effect: Preparation, Properties and Applications. Isr. J. Chem. 2011, 51, 36–55. 10.1002/ijch.201000057.

[ref67] Bian Y.; Gu Y.; Zhang X.; Chen H.; Li Z. Engineering BiOBr I1 Solid Solutions with Enhanced Singlet Oxygen Production for Photocatalytic Benzylic C H Bond Activation Mediated by N-Hydroxyl Compounds. Chin. Chem. Lett. 2021, 32 (9), 2837–2840. 10.1016/j.cclet.2021.02.006.

[ref68] Ren K.; Liu J.; Liang J.; Zhang K.; Zheng X.; Luo H.; Huang Y.; Liu P.; Yu X. Synthesis of the Bismuth Oxyhalide Solid Solutions with Tunable Band Gap and Photocatalytic Activities. Dalton Trans. 2013, 42 (26), 9706–9712. 10.1039/c3dt50498k. 23680910

[ref69] Li J.; Pei X.; Wang Z.; Li Y.; Zhang G. Boosted Charge Transfer and Selective Photocatalytic CO2 Reduction to CH4 over Sulfur-Doped K0.475WO3 Nanorods under Visible Light: Performance and Mechanism Insight. Appl. Surf. Sci. 2022, 605, 154632 10.1016/j.apsusc.2022.154632.

[ref70] Wu X.; Li Y.; Zhang G.; Chen H.; Li J.; Wang K.; Pan Y.; Zhao Y.; Sun Y.; Xie Y. Photocatalytic CO 2 Conversion of M 0.33 WO 3 Directly from the Air with High Selectivity: Insight into Full Spectrum-Induced Reaction Mechanism. J. Am. Chem. Soc. 2019, 141 (13), 5267–5274. 10.1021/jacs.8b12928. 30832477

[ref71] Wang X.; Wang Z.; Li Y.; Wang J.; Zhang G. Efficient Photocatalytic CO2 Conversion over 2D/2D Ni-Doped CsPbBr3/Bi3O4Br Z-Scheme Heterojunction: Critical Role of Ni Doping, Boosted Charge Separation and Mechanism Study. Appl. Catal., B 2022, 319, 121895 10.1016/j.apcatb.2022.121895.

[ref72] Feng X.; Zheng R.; Gao C.; Wei W.; Peng J.; Wang R.; Yang S.; Zou W.; Wu X.; Ji Y.; Chen H. Unlocking Bimetallic Active Sites via a Desalination Strategy for Photocatalytic Reduction of Atmospheric Carbon Dioxide. Nat. Commun. 2022, 13 (1), 2146 10.1038/s41467-022-29671-0. 35443754 PMC9021305

[ref73] Xu H.-Y.; Han X.; Tan Q.; He X.-L.; Qi S.-Y. Structure-Dependent Photocatalytic Performance of BiOBr_x_I_1-x_ Nanoplate Solid Solutions. Catalysts 2017, 7 (5), 153 10.3390/catal7050153.

[ref74] Ren X.; Li J.; Cao X.; Wang B.; Zhang Y.; Wei Y. Synergistic Effect of Internal Electric Field and Oxygen Vacancy on the Photocatalytic Activity of BiOBrxI1–x with Isomorphous Fluorine Substitution. J. Colloid Interface Sci. 2019, 554, 500–511. 10.1016/j.jcis.2019.07.034. 31326783

