# Supplementary material for: Visible-Light-Active Iodide-Doped BiOBr Coatings for Sustainable Infrastructure
Source: ACS Appl Mater Interfaces. 2023 Oct 12;15(42):49270–80. doi: 10.1021/acsami.3c11525 (PMC10614188; doi:10.1021/acsami.3c11525)
Supplement: Supplementary file 1 — am3c11525_si_001.pdf [file am3c11525_si_001.pdf]

Supporting Information

Visible-light active iodide-doped BiOBr coatings for sustainable infrastructure

*Mingyue Wang,[a] Raul Quesada-Cabrera,[a,b] Sanjayan Sathasivam,[a, c] Matthew O. Blunt,[a] Joanna Borowiec,[a] Claire J. Carmalt[a]**

AUTHOR ADDRESS

[a] Department of Chemistry, University College London, 20 Gordon Street, London WC1H 0AJ, UK.

[b] Department of Chemistry, Institute of Environmental Studies and Natural Resources (i-UNAT, FEAM), Universidad de Las Palmas de Gran Canaria, Campus de Tafira 35017, Spain.

[c] School of Engineering, London South Bank University, London SE1 0AA, UK.

*Corresponding author e-mail: c.j.carmalt@ucl.ac.uk

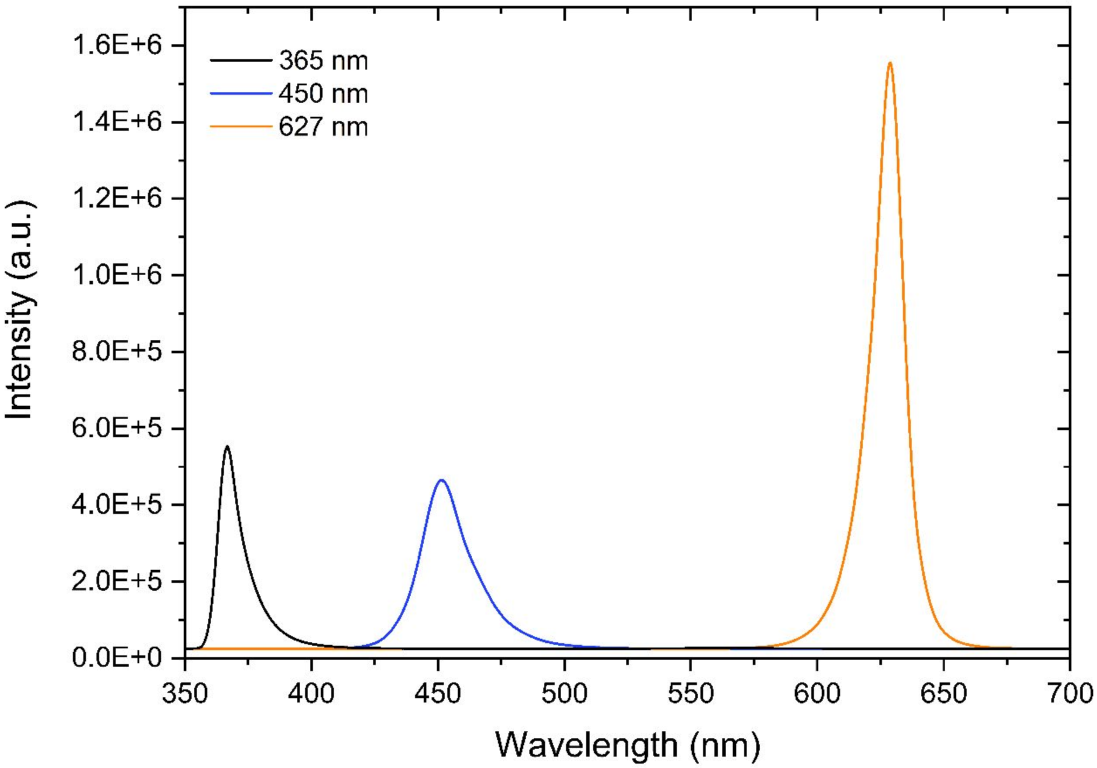

Figure S1. The emission spectra of the 365nm, 450 nm and 627 nm LED.

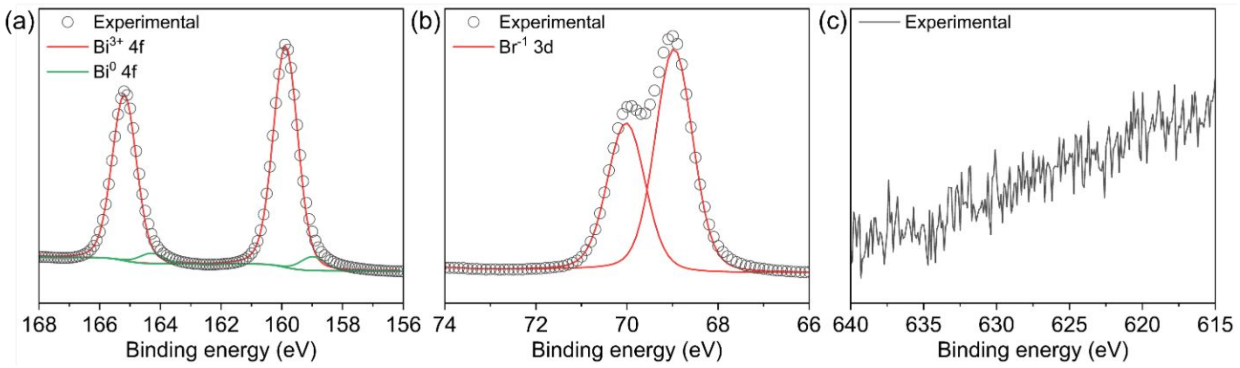

Figure S2. High resolution XPS spectra of the (a) Bi 4f and (b) Br 3d and (c) I 3d peaks from the BiOBr film.

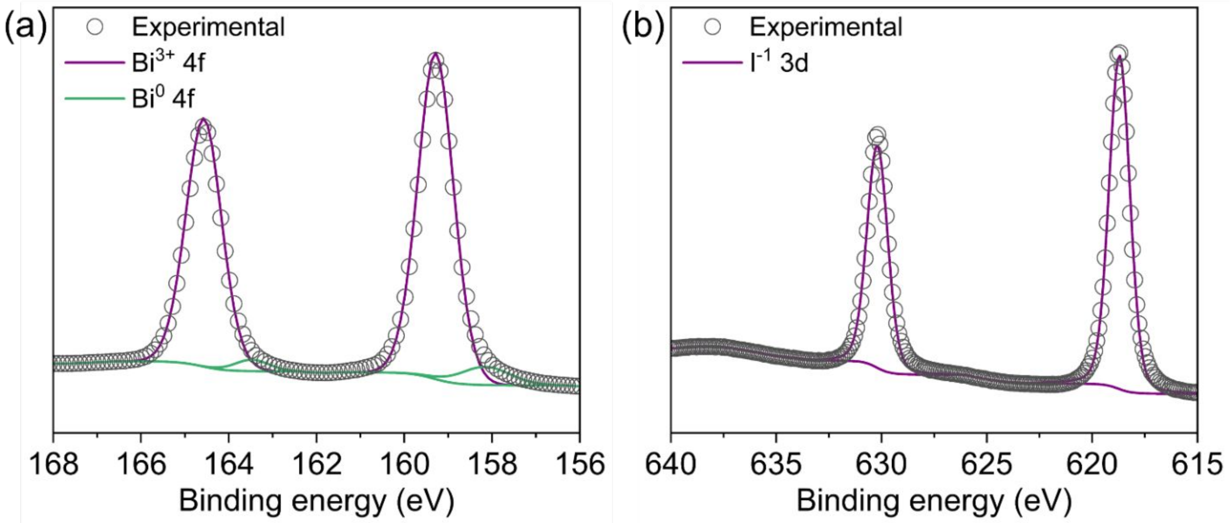

Figure S3. High resolution XPS spectra of the (a) Bi 4f and (b) I 3d peaks from the BiOI film.

Table S1. Unit cell parameters (a, c), cell volume (V) and average crystallite size of BiOBr, BiOI and **I-BB** films.

Films	a [Å]	c [Å]	V [Å^3]	Average crystallite size (Å)
BiOBr standard	3.923	8.105	124.75	-
BiOI standard	3.994	9.149	145.9	-
BiOBr	3.923 (0)	8.095 (1)	124.60 (2)	249 (4)

**I1-BB**	3.924 (0)	8.100 (1)	124.71 (2)	310 (7)
**I2-BB**	3.925 (0)	8.104 (1)	124.82 (2)	319 (7)
**I3-BB**	3.925 (0)	8.112 (0)	124.94 (0)	313 (8)
**I4-BB**	3.926 (1)	8.117 (3)	125.09 (6)	337 (10)
**I5-BB**	3.924 (0)	8.129 (7)	125.18 (11)	293 (5)
**I6-BB**	3.925 (1)	8.144 (2)	125.44 (5)	293 (4)
**I7-BB**	3.926 (1)	8.167 (2)	125.86 (5)	274 (7)
BiOI	3.996 (1)	9.148 (8)	146.10 (14)	178 (4)

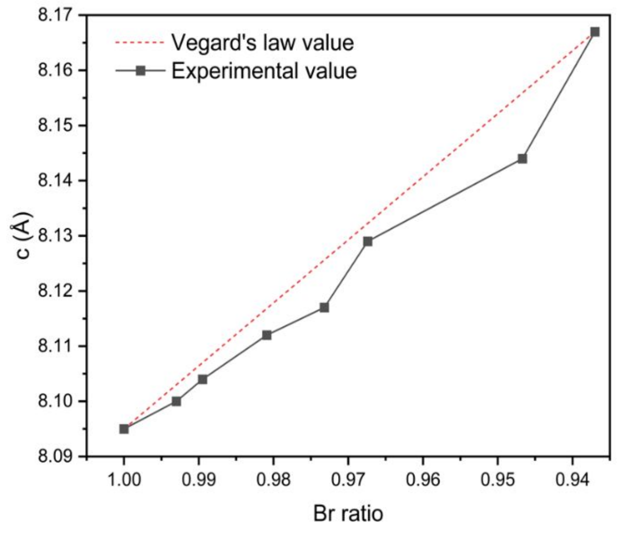

Figure S4. Comparison of *c* values of **I-BB** samples calculated from GIXRD patterns and from the Vegard's law.

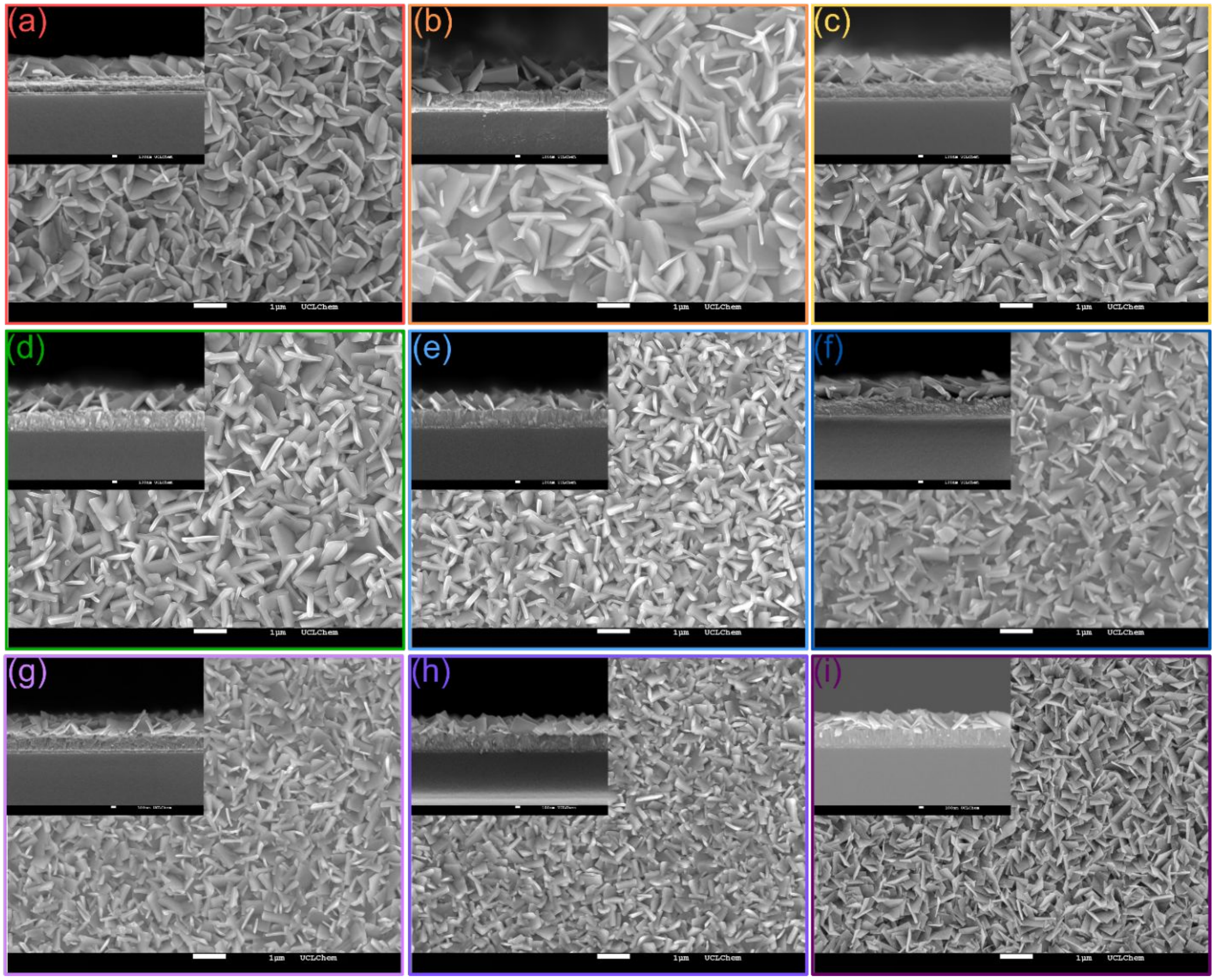

Figure S5. Top-down SEM and inserted cross-sectional SEM images of (a) BiOBr, (b) **I1-BB**, (c) **I2-BB**, (d) **I3-BB**, (e) **I4-BB**, (f) **I5-BB**, (g) **I6-BB**, (h) **I7-BB** and (i) BiOI.

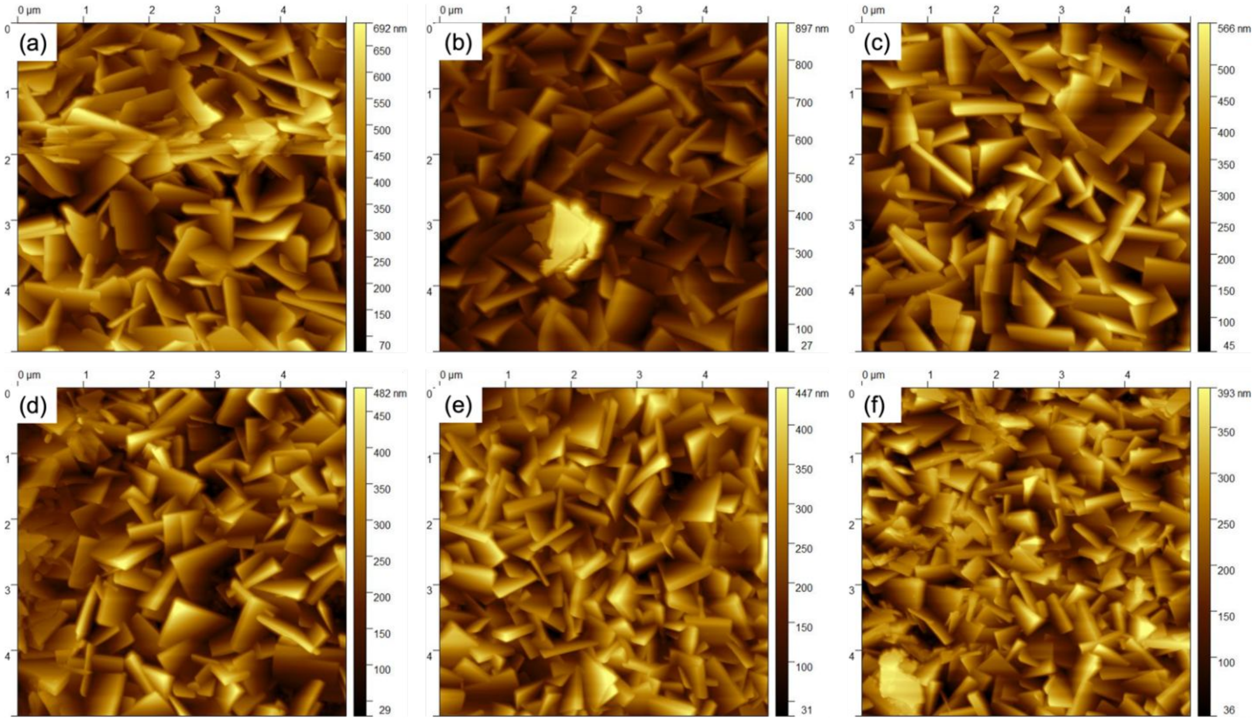

Figure S6. AFM images of (a) **I1-BB**, (b) **I2-BB**, (c) **I3-BB**, (d) **I5-BB**, (e) **I6-BB** and (f) **I7-BB** films. All images were of a 5 x 5 µm square area.

Table S2. Roughness factors of BiOBr, **I-BB** and BiOI films.

	BiOBr	**I1-BB**	**I2-BB**	**I3-BB**	**I4-BB**	**I5-BB**	**I6-BB**	**I7-BB**	BiOI
Roughness factor	2.03	1.87	1.83	1.70	1.67	1.54	1.48	1.52	1.76

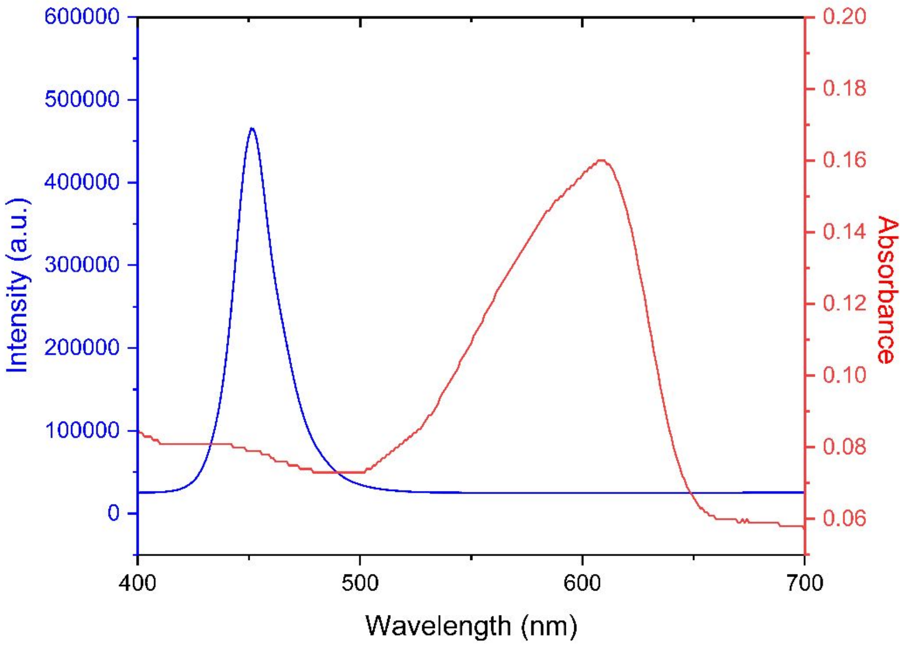

Figure S7. The absorption spectrum of the *Rz* smart ink coated on blank FTO. The blue lines illustrate the emission spectra of the 450 nm LED.

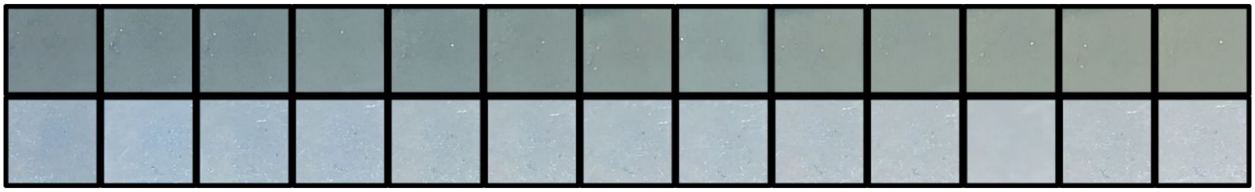

Figure S8. Images of the ink coating on **I5-BB** (upper) and FTO (lower) under 627 nm irradiation. Images were taken every 2 mins.

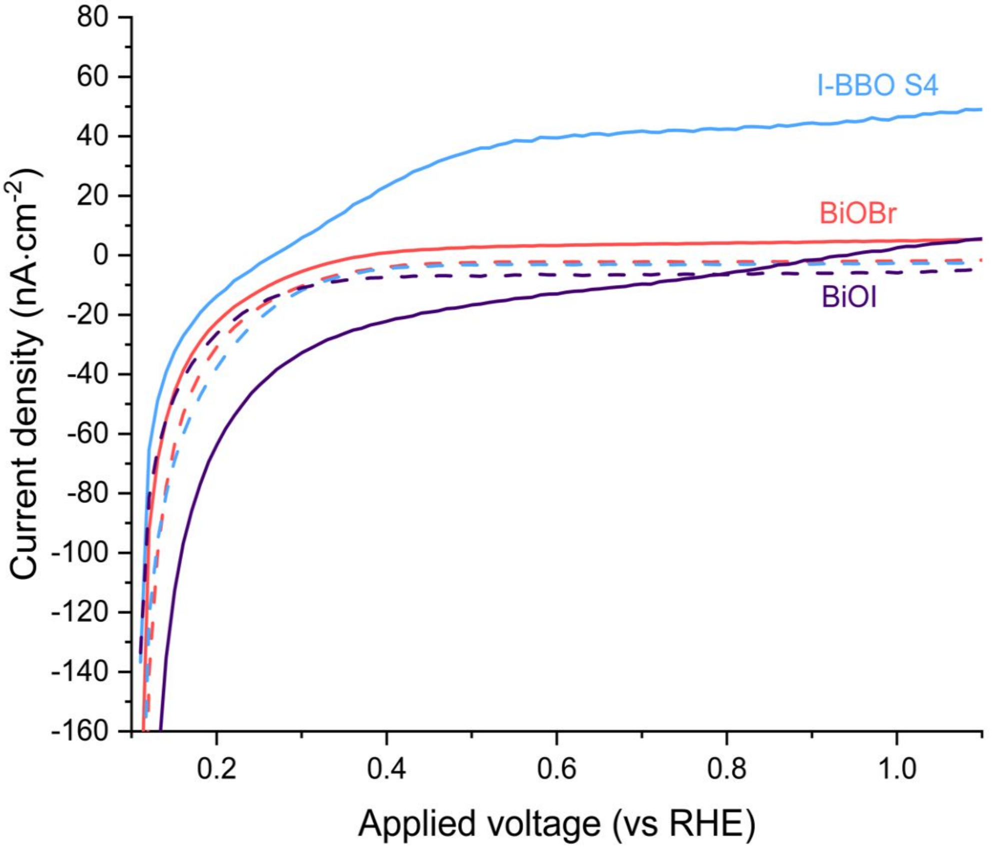

Figure S9. Current density−voltage curves of BiOBr, **I4-BB** and BiOI films on FTO were measured with front-side illumination (solid lines) and in the dark (dashed lines). The voltage was swept from 0.13 V_{RHE} to 1.10 V_{RHE} at a rate of 10 mV s^{-1}. A 220-W Xe lamp with a 420-nm cut-off filter was used as the light source (1 sun illumination). The electrolyte was an aqueous 0.5 M Na_2SO_4 solution at pH 6.6.
